# Ketorolac modulates Rac-1/HIF-1α/DDX3/β-catenin signalling via a tumor suppressor prostate apoptosis response-4 (Par-4) in renal cell carcinoma

**DOI:** 10.1038/s41598-023-32627-z

**Published:** 2023-04-06

**Authors:** Vinay Sonawane, Jeevan Ghosalkar, Swati Achrekar, Kalpana Joshi

**Affiliations:** Cell Biology Division, Cipla Ltd., LBS Marg, Vikhroli West, Mumbai, 400083 India

**Keywords:** Cancer, Cancer microenvironment, Cancer therapy, Urological cancer

## Abstract

Renal cell carcinoma (RCC) is the most difficult-to-treat form of kidney cancer with a median 5-year survival of 10% under metastatic setting. In RCC, although cytoreductive nephrectomy is common, approximately 20–30% of patients will develop recurrent cancer after surgery, which highlights the need for an effective therapy. Rho-GTPases viz, Rac-1 and Cdc42 are the central regulators of cancer cell migration and invasion and thus metastasis in multiple cancer types. Hence, we elucidated the role of Ketorolac, a modulator Rho-GTPases against RCC through potentiation of tumor suppressor Par-4. The effect of Ketorolac alone and in combination on proliferation, apoptosis, cell-cycle progression, migration, tumor inhibition and their related markers were studied. Moreover, Ketorolac’s impact on metastasis by influencing Rac-1/HIF-1α/DDX3/β-catenin signalling was studied with respect to its ability to modulate the expression of tumor suppressor Par-4, and this mechanism was confirmed by siRNA knockdown studies. Ketorolac induced cytotoxicity in a panel of renal cells including patient derived tumor cells with IC_50_ 2.8 to 9.02 mM and 0.28 to 3.8 mM in monolayer and anchorage independent clonogenic assays respectively. Ketorolac caused significant down regulation of proliferation (Ki-67, Cyclin D1, pRB and DDX3), migration/invasion (Rac-1, Cdc42, and Tiam1), and angiogenesis (HIF-1α and VEGF) markers as studied by gene and protein expression. Moreover, it caused a significant upregulation of tumor suppressor Par-4 known to be downregulated in RCC. This mechanism was further confirmed by using siRNA knockdown studies where we could demonstrate a negative relation between the expression of Par-4 and Rac-1/Cdc42. Importantly, Ketorolac alone and in combination with Sunitinib showed tumor growth inhibition (TGI) of 73% and 86% respectively in xenograft model. This anti-tumor activity was further corroborated by down regulation of Rac-1/Cdc42/HIF-1α/DDX3/β-catenin signalling. This is the first report which implicates the role of Ketorolac against RCC by acting as a small molecule secretagogue causing upregulation of Par-4 in autocrine and paracrine manner. Consequently, these findings suggest that Par-4 can serve as a valuable therapeutic target and a prognostic marker for the treatment of RCC.

## Introduction

Renal cell carcinoma (RCC) is the thirteenth most common cancer accounting for more than 90% of kidney tumors^1,2^. Globally, RCC encompasses ~ 2% of the total cancer diagnosed and deaths with an estimate of 400,000 new cases and 175,000 deaths per year^3–5^. RCC remains difficult-to-treat disease due to distinct tumor heterogeneity owing to multiple subtypes of diverse molecular features unveiling poor clinical outcome^6^. Historically, metastatic RCC (mRCC) was regarded as refractory to any adjuvant therapy if scope for surgery is limited. Notably, surgical excision of localized tumor offered significant survival benefit, but inadequate treatment options for mRCC posed dismal survival rate (5-year) of 12%^3^. Despite significant advancement in the treatment options of chemotherapy including targeted therapies and immunotherapies, mRCC remains an incurable disease until now and there is much to expect from the treatment of RCC^7,8^. Multiple factors like genetic mutations, epigenetic modifications, intratumor heterogeneity and aberrant signalling pathways collectively contribute for relapse and recurrence^9–11^. Due to the absence of effective therapy in the clinic, there is a high unmet medical need for better treatment options, particularly for high-risk mRCC patients. Considering high cost (~ 1.8 billion USD) and long-time frame (13.9 Y) required for de novo drug development, repurposing of drugs could be better option for speedy clinical impact at low cost and time^12,13^.

In this study, we report repositioning of Ketorolac, a first-generation FDA approved nonsteroidal anti-inflammatory drug (NSAID) as a potential candidate against RCC. Ketorolac is a chiral molecule administered as 1:1 racemic mixture of S and R-enantiomers. Ketorolac is recommended for short-term management of moderately severe acute pain, particularly in a postoperative setting^14^. The anti-inflammatory action of Ketorolac is attributed to the S-enantiomer which modulates the prostaglandin pathway through competitive inhibition of cyclooxygenase (COX) but lacks anti-cancer activity. The R-enantiomer on the other hand inhibits Rac-1 and Cdc42 with IC_50_ of 0.57 and 1.07 µM respectively and does not have COX inhibitory activity^15^. NSAIDs are investigated for their possible anticancer activity in breast^16^, prostate^17^, colorectal^18^, ovarian^19^ and head and neck cancer^20^. NSAIDs might play a pivotal role in the anticancer effects due to crosstalk of signaling pathways and common molecular targets shared in both, inflammation and cancer^21^. Interestingly, recently Ketorolac has been shown to promote long-term survival in animal models of cancer metastasis when administered before surgery^22^. Ketorolac is also reported to be associated with improved survival in cancer patients^23^ and effective for pain associated with cancer that has metastasized to bones^24^. The use of Ketorolac at the time of surgery to improve clinical outcome has been tested in a randomized clinical trial^19^. It has been reported that Ketorolac provided early survival benefit to breast^25^, ovarian cancer^26–28^ and NSCLC patients^29^. The pharmacological role of Ketorolac in cancer is best viewed through two distinct classes of targets COX and Rac-1/Cdc42, which may contribute to the anti-inflammatory and anti-cancer activity. Thus, Ketorolac holds promise to be rapidly translated to bedside for the treatment of mRCC alone or in combination. However, the anticancer potential of Ketorolac is poorly explored for its molecular mechanism and warrants further investigation. Here we report for the first time the efficacy of Ketorolac in difficult-to-treat RCC and its novel mechanism of action as a potent Par-4 secretagogue. Par-4, a tumor suppressor is down regulated in several cancers including RCC, conferring resistance to treatment therapy^41^. Additionally, it is reported that replenishment of low levels of Par-4 in primary renal cell carcinoma cultures sensitizes the cells to apoptosis by chemotherapeutic agents^30^. Evidently, down regulation of Par-4 in RCC patients further warrants use of Ketorolac as a potential candidate in RCC. Here we demonstrate that Ketorolac being a potent secretagogue causing Par-4 induction leads to modulation of Rac-1/Cdc42 thereby inhibiting proliferation, hypoxia, metastasis and apoptosis in RCC. Taken together, compelling evidence generated during this study suggest that Ketorolac could be mechanistically suitable candidate to treat resistance/recurrence in RCC. We believe inhibition of multiple pathways and modulation of tumor suppressors could provide insight into potentially active drug combinations for future treatment of mRCC.

## Materials and methods

### Cell lines and reagents

The renal cell lines viz, 786-O, A-498, Caki-1 and SN12C were procured from ATCC and cultured in ATCC recommended complete media. ACHN (ECACC 88100508) cells were procured from European Collection of Cell Cultures, Salisbury, UK. Experimental methods on established patient derived xenograft (PDX) cells, RXF-1183L, RXF-1220L, RXF-1781L, RXF-393L and RXF-486L were conducted at Charles River, Germany. Atg++ (Mouse embryonic fibroblast cells) were procured from University of Kentucky and were cultured in DMEM media. The cells were maintained in a humidified chamber at 37 °C and 5% CO_2_. All the compounds were dissolved in dimethyl sulfoxide (DMSO, #TC185, HiMedia) at a concentration of 10 mM and diluted in culture medium immediately before use. Ketorolac (#VIA-801223), Sunitinib malate (#SU0011214) and Sorafenib tosylate (#1615876) were procured from Chemical Centre (Mumbai, India). Dulbecco’s phosphate buffered saline (DPBS) without calcium and magnesium (#14025092), DMEM (#10566016), IMDM (#21980) and FBS (#26140079) were purchased from Gibco. Lookout mycoplasma PCR kit (#MP0035) and PMS (#P9625) were from Sigma while 96-well flat-bottomed white polystyrene plates were from Corning (#3912) and MTS reagent from Promega (#G111).

### In vitro cytotoxicity in 2D cell monolayer

In vitro anti-tumor activity of Ketorolac was evaluated in a panel of RCC lines using Cell Titer-Blue® cell viability assay. Cells were seeded in 96 well-plates (4000–10,000 cells/well), kept at 37 °C at 5% CO_2_. Post 24 h incubation, Ketorolac was added in the wells at indicated concentrations in triplicate for 72–96 h. After incubation, spent media was replaced with 100 µL fresh media and 20 µL of MTS: PMS reagent or Cell Titer-Blue reagent was added to the wells. Cells were incubated for ~ 4 h and formation of colored tetrazolium product was measured at 490 nm or relative fluorescence (RFU) was measured at Ex/Em:570/600 using Spectramax ID5 plate reader (Molecular devices, USA). The results were calculated as % T/C over control. Half-inhibitory concentration (IC_50_) values were computed with the help of GraphPad Prism using 4-parameter sigmoidal non-linear curve^31^.

### Ex vivo 3D cytotoxicity assay

Ketorolac was also tested for its ability to inhibit growth of PDX or renal cell lines in an anchorage independent semi-solid soft agar assay. The assay plates were prepared by layering 50 µL of semi-solid agar containing 3000 cells/well in ultra-low attachment plates. A second layer of 100 μL medium was added followed by 24 h incubation. Post 24 h test compound was added after serial dilution in IMDM. Cultures were incubated at 37 °C and 7.5% CO_2_ in a humidified atmosphere for 8 or 13 days and monitored closely for colony growth using an inverted microscope. This led to the formation of colonies measuring diameter more than 50 μm. At the time of maximum colony formation, counts were measured with an automatic image analysis system (Cell Insight NXT, Thermo Scientific). Viable colonies were stained using INT (Iodonitrotetrazolium Chloride)^31^.

### Ex vivo combination studies

Cytotoxic effect of Ketorolac and Sunitinib or Sorafenib was tested alone or in combination to inhibit anchorage-independent ex vivo tumor formation in semi-solid agar medium. Experiments were performed using RXF-1183, RXF-486, 786-O, A-498 and SN12C cells in a 5 × 5 combination matrix. The effect of drug combination was evaluated using bliss independent analysis method to determine index of synergy.

### Colony forming assay

A-498, 786-O and Caki-1 cells were seeded in 6-well plates (500 cells/well) and incubated in complete media for 24 h. Cell were treated with Ketorolac (1, 3 and 5 mM) and Sunitinib (4 µM) alone or in combination. After 48 h incubation, media containing drugs was replaced with fresh media and plates were further incubated for 7–10 days. The colonies formed were washed with 1 × PBS. Colonies were fixed with methanol: acetic acid mixture (1:6) for 30 min followed by washing and then by staining with 0.5% crystal violet for 30 min. Visible colonies were quantified using Image J software (V1.52a).

### Cell cycle analysis

Effect of Ketorolac on RCC cells was analyzed for ploidy content using flow cytometry. A-498 cells were treated with Ketorolac at indicated concentrations alone or in combination with Sunitinib. After treatment, cells were trypsinized, centrifuged at 400×*g* for 10 min and washed twice with cold PBS. Further, cells were fixed with ice chilled 70% ethanol at 25 °C for 30 min. Ethanol fixed cells were centrifuged at 1000 rpm for 5 min and washed twice with PBS. Finally, cells were resuspended in PBS containing RNase A and propidium iodide (PI) and incubated at 25 °C for 45 min. Ten thousand cells were acquired using flow cytometer (BD FACS Lyric) followed by data analysis on BD FACSuite software. The analysis area was gated for selection of cell population. The peak channels were marked and % population obtained for each phase of the cell cycle^31^.

### Wound healing assay

A-498 cells were seeded in 24-well plates (1 × 10^6^ cells/well) and incubated at 37 °C, 5% CO_2_ for 24 h. Once 80–90% confluency was achieved, scratches were inflicted using micropipette tip in each well. To remove the detached cells, plates were washed using PBS and subsequently, they were incubated with serum-free medium containing Ketorolac (1, 3 and 7 mM) and Sunitinib (4 µM) alone or in combination for 24 h. Distance between the edges of the wound was measured 24 h post drug addition. Images were captured at 10×. Captured images were further quantified using Image J software to measure closure of the wound.

### Protein expression studies

Protein expression was analyzed by Western blotting. Cells were harvested in 200 µL of 2× Lamelli buffer (# ML021, HiMedia, India), heat denatured at 100 °C and snap-chilled on ice. Total protein content in the lysates was estimated using Pierce TM BCA protein assay kit. (#23227, Thermofisher, MA, USA). Cell lysate proteins (~ 50 μg) were separated on 7.5–12% SDS polyacrylamide gels by electrophoresis and transferred by dry method to a PVDF/nitrocellulose membrane using iblot Dry Blotting System (#IB21001, Thermofisher, MA, USA). The membranes were blocked with non-fatty milk (#1706404, Bio-Rad, CA, USA) for an hour at RT followed by 3 washes with PBS containing 0.1% Tween (PBST). Further, membranes were incubated with various primary antibodies Par-4 (#R-334, Santa Cruz Biotech, TX, USA), Cdc42 (#2462S, Cell Signaling, MA, USA), pRB (#D20B12 Ser807/811, Cell Signaling, MA, USA), DDX3 (#2635S, Cell Signaling, MA, USA), Tiam1 (#PS5-80130; Thermo Fisher, MA, USA), Rac-1 (#2465, Cell Signalling, MA, USA), Cyclin D1 (#2922, Cell Signaling, MA, USA) and GAPDH (#MA5-15738, Invitrogen, Canada) was used as a housekeeping control. The membranes were incubated with respective secondary antibodies (1:10,000 or 1:20,000) for 1 h at RT. After further 3 washes with PBST, the immunoblots were developed using enhanced luminol-based chemiluminescent substrate (ECL) substrate and the images were visualized using the ChemiDoc XRS System (Version 6.1, Bio-Rad, CA, USA). Images were further quantified for densitometry analysis using Image J software.

### Gene expression

A-498 cells were seeded in 6-well plates (1.0 × 10^6^ cells/well) for 24 h. The media was replaced with fresh media and the cells were treated with Ketorolac (1, 3 and 7 mM) and Sunitinib (4 µM) alone or combination at indicated concentrations. Cells were harvested at different time points (2, 6, 18 and 24 h) and RNA was extracted using Trizol (#15596026, Invitrogen, Carlsbad, Canada). The first-strand cDNA was synthesized (2 μg of total RNA) using Superscript III reverse transcriptase enzyme (Invitrogen, Carlsbad, Canada) and oligo deoxythymidine (dT) as primers (Sigma Aldrich, India). The aliquots of amplified products of RT-PCR reaction were run on electrophoresis using 1.5% agarose gel (#MB080, HiMedia, India) with 7 μL hi-Syber safe dye (#ML053, HiMedia, India) in 1× TBE buffer (#ML016, HiMedia, India) at 100 V/20 min along with a PCR marker (#MBT049, HiMedia, India). Gel images were obtained using Bio-Rad Doc system and quantified for densitometry analysis using Image J software. Quantitative real time PCR (qRT-PCR) was performed using SYBR Green Supermix (Thermofisher, MA, US) as per the manufacturer’s instructions. The relative changes in mRNA expression levels were assessed by 2^−ΔΔCT^ method and changes in mRNA expression of target gene were normalized to that of GAPDH gene. The primer pairs of selected genes are listed in Additional File 1, Fig. 1.

### siRNA mediated silencing of Par-4 and Rac-1

A-498 cells (2.5–5 × 10^5^ cells/well) were plated in six-well plates in complete media. The cells were transfected with 30 pmol of Par-4 siRNA (#S10049, Thermofisher, MA, USA)^32^ and Rac-1 siRNA (#S11712, Thermofisher, MA, USA) using Lipofectamine RNAiMAX transfection reagent (#4392420, Invitrogen, MA, USA) as described by the manufacturer. The siRNA–Lipofectamine complex was added dropwise to the cells and incubated at 37 °C for 6 h. Post transfection, media was replaced with complete DMEM medium and the cells were harvested at 2, 5, 18, and 24 h for mRNA expression and 24, 48 and 72 h for protein expression of Par-4 (#R-334, Santa Cruz Biotech, TX, USA) and Rac-1 (#2465, Cell Signaling, MA, USA) and GAPDH served as a loading control (#MA5-15738, Invitrogen, MA, USA).

### In vivo efficacy

Athymic nude mice (female, ~ 6–8 weeks) from Vivo Bio Tech Ltd, Hyderabad, India were housed in IVC cages with 12 h light & dark cycle, 55–75% relative humidity at 22–25 °C. Animal studies were carried out in accordance with relevant guidelines and regulations laid by Committee for the Purpose of Control and Supervision of Experiments on Animals (CPCSEA, EP022/16 and 01-2017). The study was conducted in accordance with Animal Research: Reporting of In Vivo Experiments (ARRIVE) guidelines. Animals were given access to autoclaved Nutrilab Rodent Feed diet (Provimi Animal Nutrition India Pvt. Ltd., Bangalore, India) and sterile water ad libitum. Mice were acclimatized for a week before tumor implantation. Mice were implanted subcutaneously with ~ 30 mg A-498 tumor fragment using Trocar needle in the right flank. All the animals were monitored for tumor growth. Once tumors attained desired size (~ 100 mm^3^), the experimental animals were selected, randomized and grouped (n = 7). CMC (0.5% in water, 10 mL/kg, PO) was used as vehicle control, Ketorolac was dosed at 5 and 10 mg/kg (mpk), Sunitinib was administered at 20 mg/kg, animals dosed with combination drugs received 5 mg/kg Ketorolac + 20 mg/kg Sunitinib or 10 mg/kg Ketorolac + 20 mg/kg Sunitinib. Daily cage side observations were made for clinical signs. Body weight and tumor volume were recorded twice weekly throughout the experimental period (Additional File 1, Fig. 2). Mean tumor volume was calculated from tumor measurement data and Tumor Growth Inhibition (TGI) was calculated from tumor volume. Pharmacodynamics markers viz, *HIF-1α, DDX3, Rac-1, Cdc42 and β-catenin* were also evaluated from the tumor fragment.

### Statistical analysis

Statistical differences between the experimental groups were determined by One-way analysis of variance (ANOVA) followed by post hoc multiple variances using a Tukey test (IBM SPSS Statistics, V20). Data computation and analysis was performed using GraphPad Prism software, Version 4.01. Efficacy of different drugs alone/combinations is expressed as modelled T/C value, which is the actual measured T/C values on a scale ranging from 0 to 1, where 1 corresponds to a T/C value of 100%. The bliss neutral value is the product of modelled T/C of individual drugs concentrations. The difference between bliss neutral value and the modelled T/C value for the various combination was taken as bliss index. Bliss index is arranged on a scale ranging from − 1.0 to 1.0. Positive values (Bliss Index ≥ 0.15, blue) indicate synergy, negative values (Bliss Index ≤  − 0.15, red) indicate antagonism, and zero is the neutral value (white)^31^.

### Ethics approval and consent to participate

The animal study protocol was approved by Institutional Ethics Committee (IEC) and Committee for the Purpose of Control and Supervision of Experiments on Animals (CPCSEA) of (EP022/16 and 01-2017).

## Results

### In vitro cytotoxicity in PDX cells and RCC cell lines

**Figure 1:**
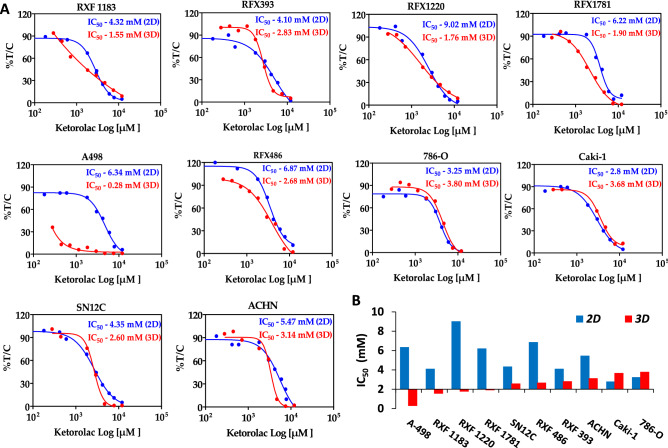
Cytotoxicity of Ketorolac in a panel of RCC cell lines. (**A**) In vitro and ex vivo cytotoxicity assay for Ketorolac in panel renal cells with indicated concentrations. Ex vivo assay was performed in anchorage independent semi-solid soft agar 3D assay using renal tumor xenograft-derived cell suspension or renal cell lines. Cytotoxicity is expressed as % survival (% T/C) as a ratio of treated by control. (**B**) Histogram represents comparison of in vitro and ex vivo activity of Ketorolac across the panel of 10 RCC cell lines.

The anti-tumor activity of Ketorolac was evaluated in a panel of ten RCC lines with diverse genetic background (Additional File 1, Table 1). These cell lines included PDX cells (RXF-1183L, RXF-1220L, RXF-1781L, RXF-393L and RXF-486L) and ATCC/ECACC cell lines (786-O, A-498, ACHN, Caki-1 and SN12C). All these cell lines were tested for IC_50_ determination in 2D monolayer and 3D anchorage-independent clonogenic assay performed in semi-solid agar medium. Ketorolac showed absolute IC_50_ values in PDX cell lines in the range of 4.12–9.02 mM in 2D assay and 1.55–2.83 mM in clonogenic assay. While IC_50_ for Ketorolac in cell line-derived models were in the range of 2.8–5.47 mM in monolayer assays as compared to 0.28–3.8 mM in clonogenic assay (Fig. 1A). Amongst all the cell lines tested for cytotoxicity of Ketorolac, A-498 (*TP53 wild* and *VHL, MLL3, SETD2* mutated), was the most sensitive (IC_50_—0.28 mM) while 786-O (*VHL, PTEN, TP53 and TSC2 mutated)* and Caki-1* (TP53 wild* and *MET, MLL3, SETD2 mutated)* showed IC_50_ value of 3.8 and 3.67 mM respectively. Overall, Ketorolac displayed a concentration-dependent activity in all cell lines with an average absolute IC_50_ value of 5.257 and 2.422 mM in 2D and 3D assay respectively. The cytotoxicity response of Ketorolac in anchorage-independent clonogenic assay was vastly better in PDX cells. PDX cells retain characteristic of parental patient tumors, therefore efficacy response is expected to be better in the clinical setting (Fig. 1B). IC_50_ values for Sunitinib and Sorafenib as a standard of care in clonogenic assay is included in Additional File 1, Table 2.

### Ex vivo combination studies

**Figure 2:**
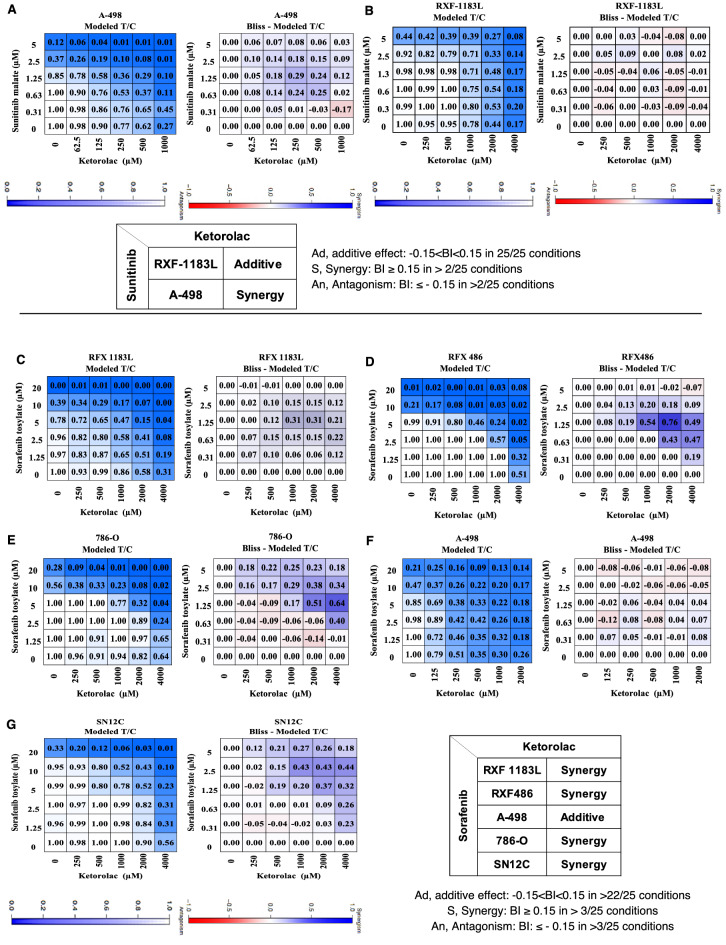
Efficacy of Ketorolac in combination with Sunitinib at indicated concentrations with 5 × 5 matrix in (**A**) RXF-1183L and (**B**) A-498 cells seeded in soft agar. Similarly, effect of Ketorolac in combination with Sorafenib was evaluated in (**C**) RFX-1183L, (**D**) RFX-486, (**E**) 786-O, (**F**) A-498 and (**G**) SN12C. Bliss independence analysis showed synergy or additive effect. The color coding of the tiles in the heatmap show that there is consistent concentration-dependent effect pointing towards synergy (BI > 0.15) or additive effect (− 0.15 < BI < 0.15).

The ability of Ketorolac to inhibit ex vivo colony formation as a single agent or in combination with Sunitinib or Sorafenib was examined in tumor cell lines of renal cancer using 3D clonogenic assay in 5 × 5 combination matrix. Ketorolac and Sunitinib inhibited colony formation of RCC cells seeded in soft agar in dose dependent manner. This is also reflected in the matrix concentration, where activity of the different combinations was observed with increasing dose of both the compounds. Bliss independence analysis showed that combination of both the drugs produced additive effect in RFX-1183L (Fig. 2A) and synergistic effect in A-498 (Fig. 2B). The color coding of the tiles in heatmap show that there is consistent effect pointing towards additive effect (− 0.15 < BI < 0.15) in > 22/25 conditions or synergy (BI > 0.15) in more than 3/25 conditions. Similarly, combination of Ketorolac with Sorafenib is synergistic in RFX-1183 (Fig. 2C), RFX-486 (Fig. 2D), 786-O (Fig. 2E) and SN12C (Fig. 2G), while additive in A-498 cells (Fig. 2F).

### Ketorolac with Sunitinib affects the clonogenicity in RCC cells

**Figure 3:**
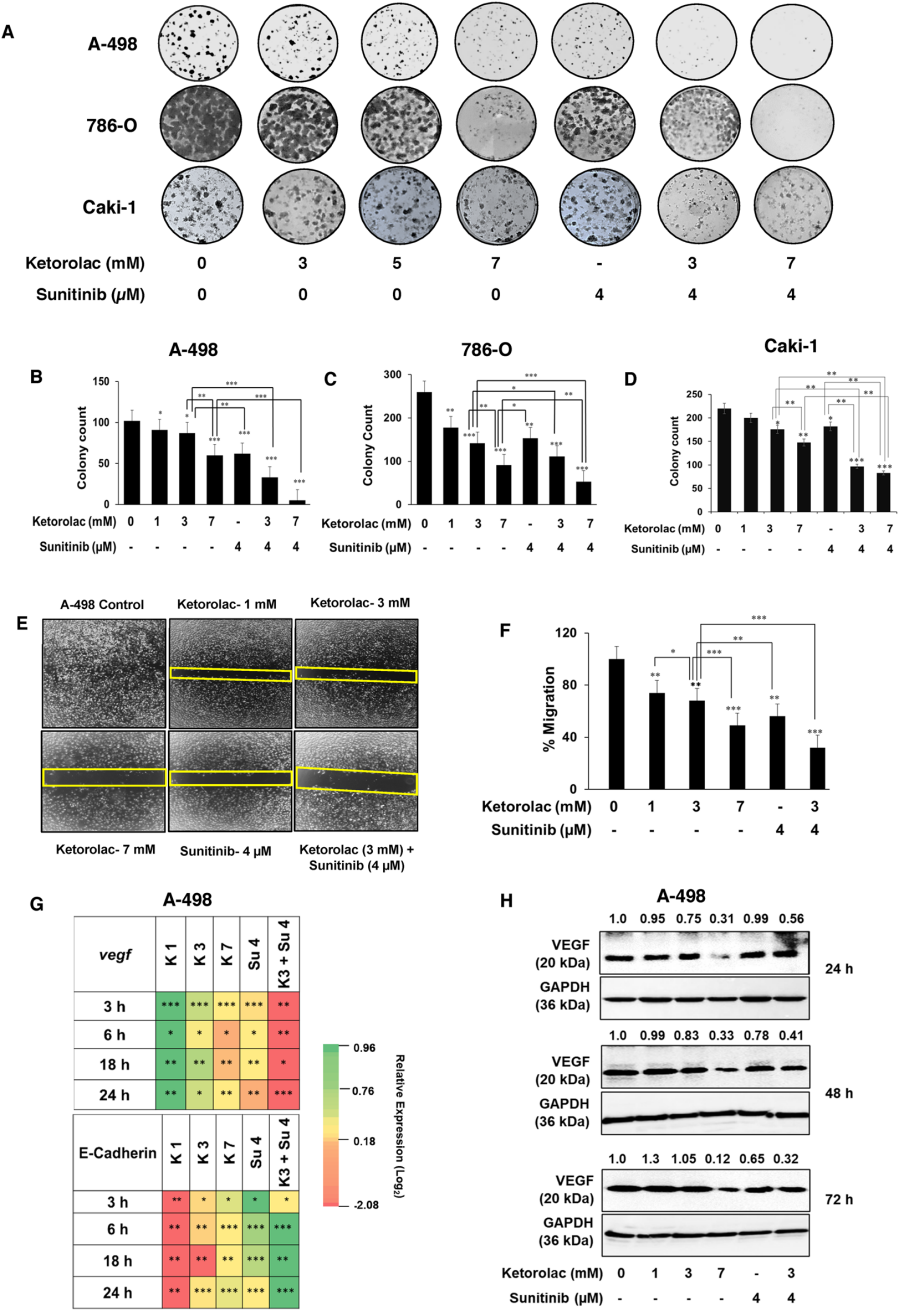
Clonogenic and wound healing assay for Ketorolac. (**A**) Representative colony pictures for A-498, 786-O and Caki-1 cells. (**B**–**D**) Show histogram representing quantitation of the stained colonies using Image J for respective cells. (**E**) Wound-healing assay in A-498 treated with Ketorolac alone or in combination with Sunitinib. (**F**) Histogram representing quantitation of images using Image J. Statistically significant difference between and within different groups was determined by one-way ANOVA and post hoc multiple variance by Tukey test (*p ≤ 0.05, **p ≤ 0.01 and ***p ≤ 0.001). (**G**) The qRT-PCR of *VEGF* and *E-cadherin* in A-498 after treatment with Ketorolac alone and in combination with Sunitinib. The gene expression was studied at 3, 6, 18 and 24 h post treatment. (**H**) The protein expression for VEGF at different time points 24, 48 and 72 h after treatment (*K* Ketorolac and *S* Sunitinib).

The effect of Ketorolac alone and in combination with Sunitinib on A-498, 768-O and Caki-1 clonogenicity was analysed (Fig. 3A). Clonogenicity of A-498, 786-O and Caki-1 cells was observed to be gradually abolished by Ketorolac both at lower and higher concentrations (1, 3, 7 mM). The combination of Ketorolac and Sunitinib drastically reduced the cell growth as compared to vehicle control and drug alone. The reduction in the clonogenicity is depicted graphically (Fig. 3B–D).

### Impairment of cell migration by Ketorolac and its combination partner Sunitinib

The effect of Ketorolac on A-498 cell migration was evaluated by wound healing assay. Cell migration was monitored over time after incubation in starvation medium with 1, 3 and 7 mM Ketorolac, 4 µM Sunitinib and combination of Ketorolac 3 mM and Sunitinib 4 µM or with vehicle. Ketorolac reduced cell migration in A-498 cells at 24 h in a dose dependent manner (Fig. 3E). It was interesting to note that the combination of Ketorolac and Sunitinib synergistically inhibited cell migration and wound closure (Fig. 3F). Importantly, Ketorolac by itself as well as in combination with Sunitinib could significantly downregulate VEGF at both transcript and protein level (Fig. 3G,H). Another notable observation was that it could also significantly upregulate the expression of E-Cadherin (Fig. 3G).

### Sequential treatment of Ketorolac followed by Sunitinib induced dose dependent apoptosis in A-498 cells

**Figure 4:**
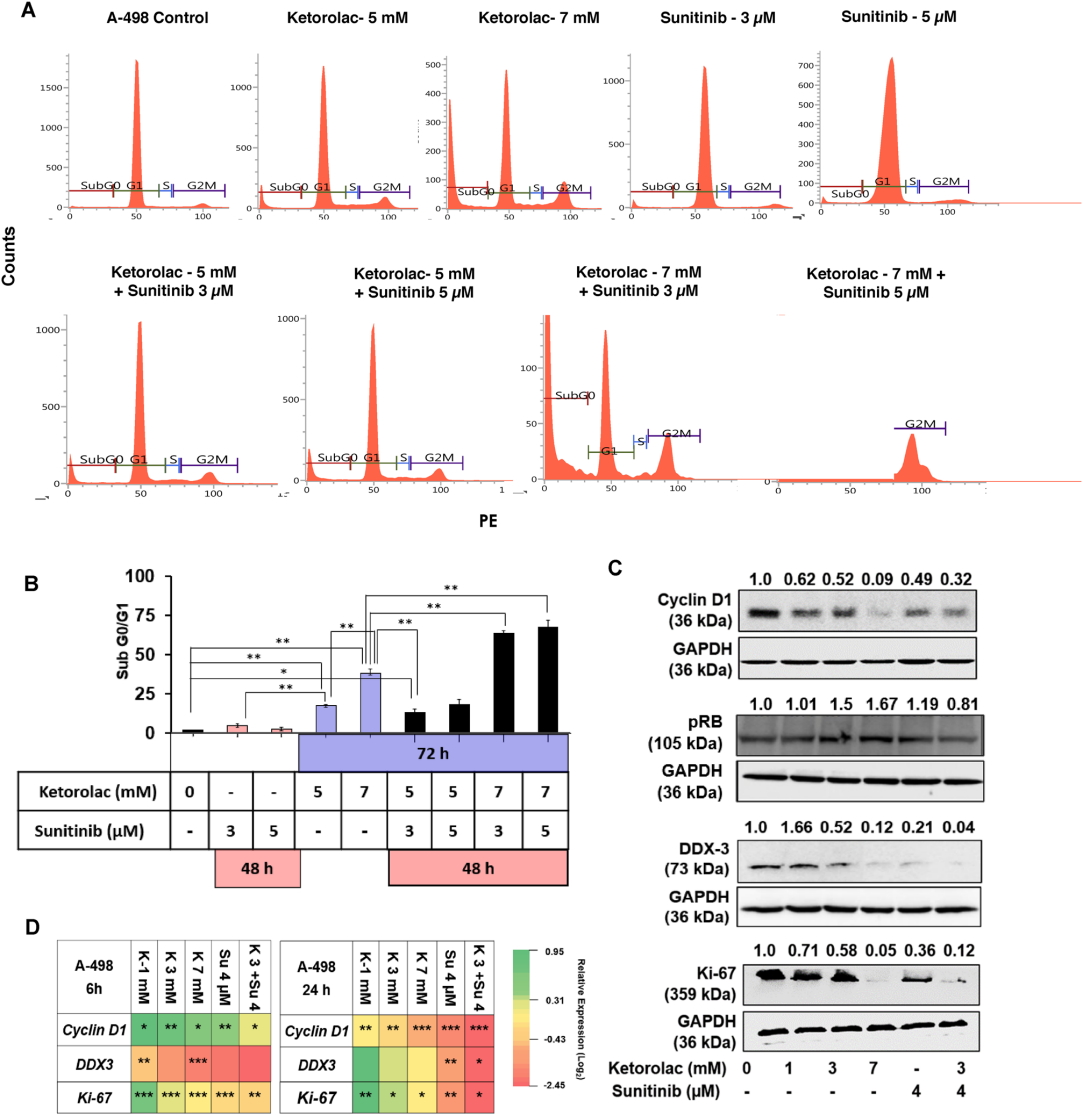
(**A**) Apoptosis effect (% sub G0/G1) in A-498 during sequence dependent Ketorolac treatment (72 h) followed by addition of Sunitinib for last 48 h. Ten thousand cells were acquired and further analyzed by BD FACS-Lyric flow cytometry. (**B**) Histograms represent increased sub G0/G1 levels of A-498 after treatment with Ketorolac followed by Sunitinib. (**C**) Western blot depicting inhibition Cyclin D1, pRB, Ki-67 and DDX3 post 24 h Ketorolac treatment and in combination with Sunitinib. (**D**) Heatmap depicts modulation of proliferation and EMT markers in A-498 at 3, 6, 18 and 24 h treatment with Ketorolac alone and in combination with Sunitinib. The relative expression of genes was calculated with the relative 2^−ΔΔCt^ method, using GAPDH as housekeeping gene for normalization (*K* Ketorolac and *S* Sunitinib).

The ability of Ketorolac to induce dose dependent apoptosis in A-498 cells was studied by cell cycle analysis using flow cytometry. Ketorolac treatment (5 and 7 mM) was given for first 24 h followed by addition of Sunitinib (3 and 5 µM) and the combination treatment was continued for total 72 h. Cells were also treated with drug alone for both Ketorolac (72 h) and Sunitinib (48 h). On termination, cells were subjected to cell cycle analysis using flow cytometer. The sequential treatment of Ketorolac followed by Sunitinb induced dose dependent increase in Sub G0/G1 levels with 13.25, 18.23, 63.95 and 67.47% for combination groups of Ketorolac 5 mM with Suntinib at 3 and 5 µM and Ketorolac 7 mM with 3 and 5 µM of Sunitinib respectively (Fig. 4A,B). This sequential addition of Ketorolac followed by Sunitinib showed a synergistic pro-apoptotic activity in A-498 cells (Fig. 4B). Importantly, Ketorolac showed a significant down regulation of important proliferation markers like Cyclin D1, pRB, DDX3 and Ki-67. This effect was seen at both transcriptional (Fig. 4D) as well as translational level (Fig. 4C). Notably, synergistic activity was observed towards inhibition of these markers by the combinaiton of Ketorolac and Sunitinib.

### Ketorolac mediated upregulation of Par-4 in normal fibroblast and A-498 cells

**Figure 5:**
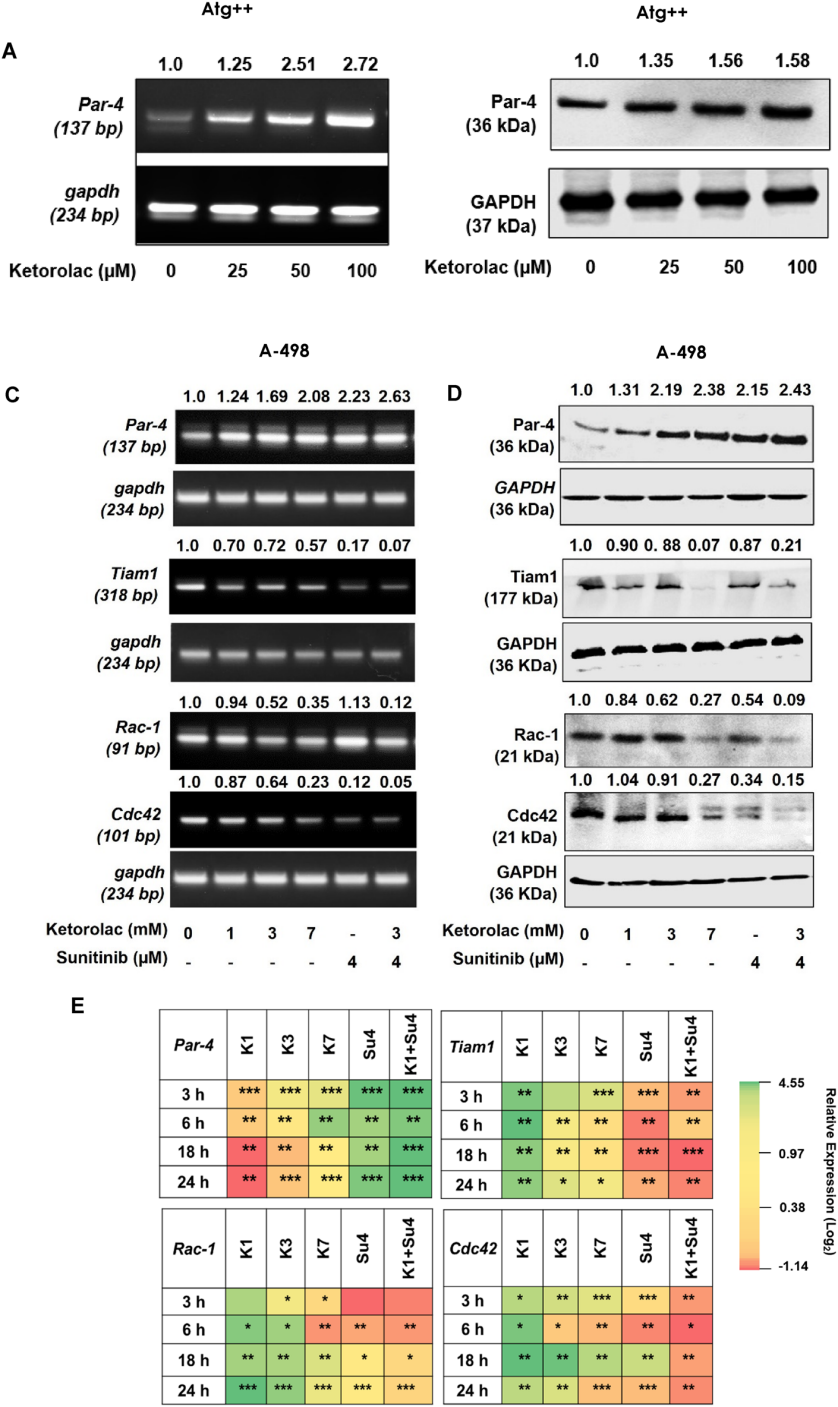
Par-4 expression in Atg++ and A-498 cells (**A**) mRNA expression of *Par-4* in Atg++ cells treated with 25, 50 and 100 µM Ketorolac for 24 h. (**B**) Par-4 protein expression in Atg++ post 48 h treatment with Ketorolac. (**C, D**) Transcript and protein expression of Par-4, Tiam1, Rac-1 and Cdc42 in A-498 cells treated with Ketorolac 1, 3, 7 mM and Sunitinib 4 µM alone and in combination for 24 h respectively. GAPDH was included as loading control. Numbers at top represent densitometric analysis of band. (**E**) Heatmap depicts modulation in transcript levels of *Par-4, Tiam1, Rac-1 and Cdc42* in A-498 at 3, 6, 18 and 24 h treatment with Ketorolac. The relative expression of genes was calculated with the relative 2^−ΔΔ^Ct method, using GAPDH as housekeeping gene for normalization (*K* Ketorolac and *Su* Sunitinib).

Par-4 being an important tumor suppressor, we checked the effect of Ketorolac on Par-4 expression at transcriptional and translational level in both normal mouse fibroblast (Atg++) and in A-498 cells. Atg++ cells were treated with 25, 50 and 100 µM Ketorolac for 24 h to analyse the expression at transcript level and for 48 h to analyze the protein expression. Ketorolac was able to cause a significant upregulation of Par-4 in a dose dependent manner in both Atg++ (Fig. 5A,B) as well as A-498 (Fig. 5C–E). Importantly, combination of Ketorolac with Sunitinib significantly down regulated the expression of important metastasis markers viz, Tiam1, Rac-1 and Cdc42 in a concentration dependent manner (Fig. 5C,D).

### Silencing of *Par-4* and *Rac-1* and modulation of genes in signalling pathway

**Figure 6:**
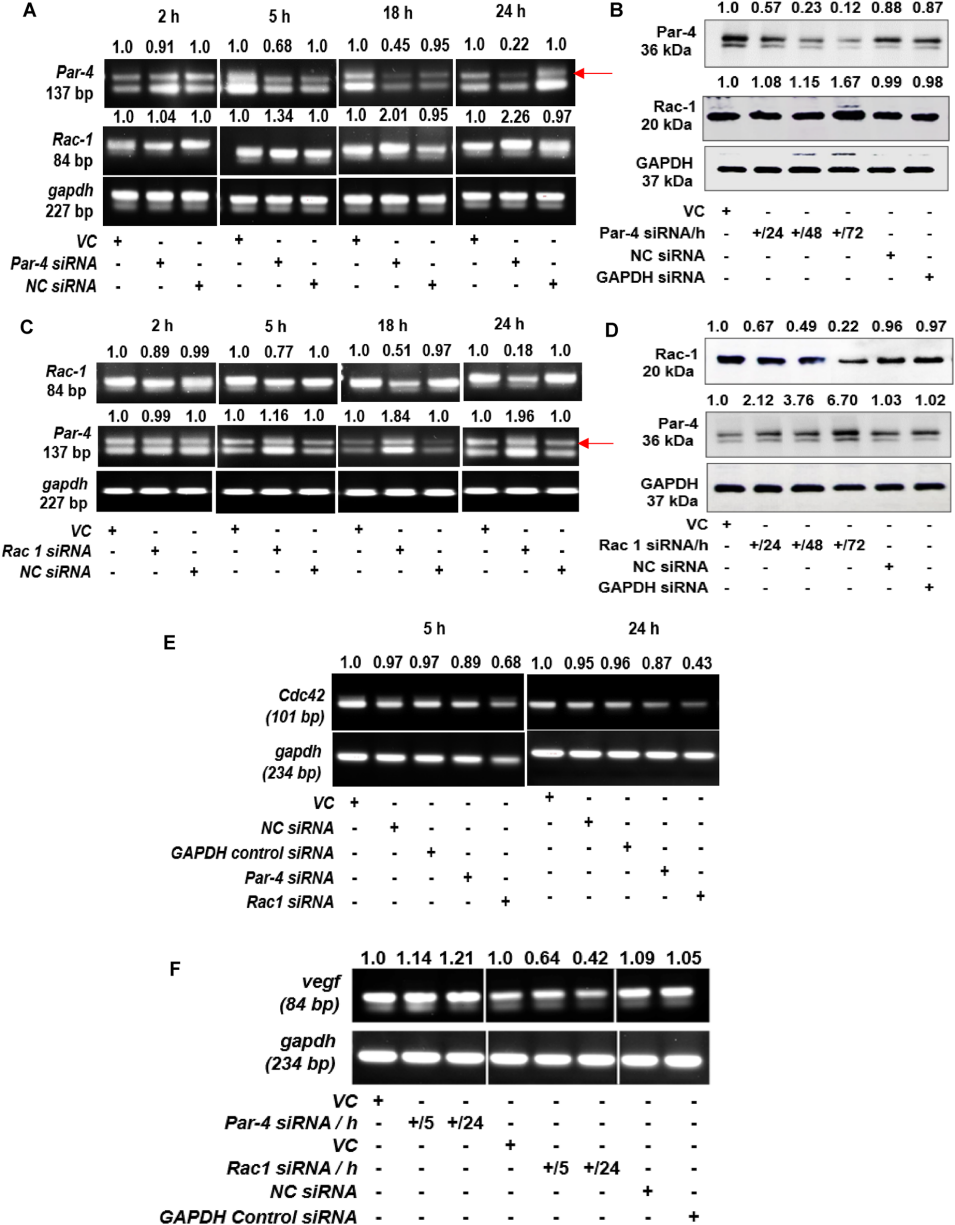
(**A, B**) Silencing of *Par-4* (30 pmol) and its effect on *Rac-1* expression in A-498 cells post 2, 5, 18 and 24 h treatment at transcript level and 24, 48 and 72 h post treatment at protein level. (**C, D**) Silencing of *Rac-1* (30 pmol) and its effect on *Par-4* expression post 2, 5, 18 and 24 h at transcript level and 24, 48 and 72 h at protein expression level. Scrambled siRNA served as a negative control. GAPDH served as a loading control. (**E**) The level of *cdc42* expression post 5 and 24 h treatment after silencing of *Par-4* and *Rac-1* independently. (**F**) The level of *VEGF* expression post 5 and 24 h after silencing *Par-4* and *Rac-1* independently. Gapdh served as a loading control. Numbers at top of the blots refer to the densitometric analysis of the immunoreactive bands and represent the fold change in gene and/or protein expression normalized to GAPDH.

To illustrate the mechanism of action of Ketorolac, A-498 cells were transiently transfected with either Par-4 siRNA (30 pmol), or Rac-1 siRNA (30 pmol), negative control siRNA or GAPDH siRNA. Post silencing, expression of Par-4 and Rac-1 mRNA was evaluated. Effective Par-4 silencing was noted from 2 h till 24 h. Importantly, Par-4 knockdown cells showed significant upregulation in Rac-1 levels of 1.04, 1.34, 2.01 and 2.26 at 2, 5, 18 and 24 h (Fig. 6A). Further, similar observation was noted at the translational level wherein the reduction of Par-4 protein expression 0.67, 0.23 and 0.12 lead to concomitant increase in Rac-1 1.08, 1.15 and 1.67 expression at 24, 48 and 72 h respectively (Fig. 6B). Likewise, the Rac-1 mRNA transcript revealed effective silencing from 5 h onwards where Par-4 levels were seen to be increased by 24 h (Fig. 6C). The fold difference for Rac-1 protein expression was noted to be 0.67, 0.49, and 0.22 and Par-4 levels were increased significantly after Rac-1 silencing by 2.12, 3.76 and 6.70-fold respectively at 24, 48 and 72 h (Fig. 6D). Vehicle control did not show difference in the levels of Par-4 or Rac-1 at any time point. Overall results indicated Rac-1 levels were found be negatively regulated by Par-4 expression. Since, Ketorolac is a known Rac-1 and Cdc42 inhibitor along with the novel role as a Par-4 inducer that we show here for the first time, we wanted to understand the relation between Par-4, Rac-1, Cdc42 and VEGF. Therefore, we looked at the expression of Cdc42 and VEGF in Par-4 and Rac-1 silenced cells. And indeed, we deciphered that Cdc42 expression was downregulated in Rac-1 silenced cells at 5 and 24 h and Par-4 silenced cells at 24 h (Fig. 6E). Similarly, the expression of VEGF was downregulated in Rac-1 silenced cells but significantly upregulated in Par-4 silenced cells (Fig. 6F). This confirms our earlier findings, where we show Ketorolac mediated upregulation of Par-4 and simultaneous downregulation of VEGF in A-498 cells (Fig. 3G,F). Furthermore, we also validated this observation by Par-4 knockdown studies (Fig. 6F).

### In vivo efficacy

**Figure 7:**
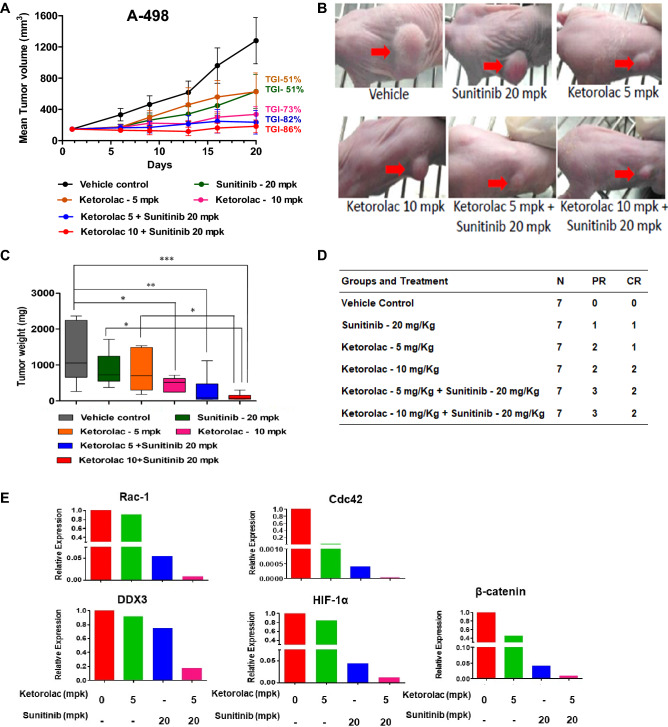
(**A**) In vivo efficacy of Ketorolac in A-498 xenograft model. Nude athymic mice were implanted with ~ 30 mg A-498 tumor fragment using trocar needle in the flank region. Once tumor reached to 100 mm^3^, animals were randomized in different groups of Ketorolac (5 and 10 mg/kg) alone and in combination with Sunitinib (20 mg/kg). Animals were weighed regularly, and tumor volume was measured every alternate day. (**B**) Representative picture of mice on the day of termination (Day 20th) form different regimen groups. (**C**) Histogram indicate tumor weight scatter plot on 20th day of the regimen. (**D**) Table shows complete and partial regression (CR and PR) of A-498 tumors in mice treated with Ketorolac alone and in combination with Sunitinib. (**E**) qRT PCR analysis in tumor samples obtained from xenograft model. The gene expression for the transcripts of *HIF-1α, DDX-3, Cdc42, Rac-1* and *β-Catenin* were studied.

In vivo efficacy of Ketorolac was performed in athymic nude mice. Interestingly, Ketorolac alone at 5 mg/kg p.o. and 10 mg/kg p.o. substantially inhibited tumor formation by up to 51% and 73% respectively. While sunitinib alone at 20 mg/kg p.o., showed 50% tumor regression. Importantly, combination of Ketorolac at 5 mg/kg p.o. or 10 mg/kg p.o. with Sunitinib at 20 mg/kg p.o. led to sufficiently enhanced inhibition of tumor formation by 82% and 86% respectively, clearly indicating superior anti-tumor efficacy as compared to drug alone (Fig. 7A–D). No adverse effect on body weights were observed indicating well tolerated dosage and schedule (Additional File 1, Fig. 2). Pharmacodynamics study performed by estimating the transcript levels of *Rac1, Cdc42, HIF-1α, β-catenin and DDX3* in the tumor tissue reveled a significant downregulation of these important metastasis markers in the combination group as compared to the drug alone group (Fig. 7E).

## Discussion

Renal Cell Carcinoma, localized as well as metastatic both show unsatisfactory responses to standard chemotherapy regimens and targeted agents in terms of disease-free survival^3^. Significant changes in the treatment landscape for patients with RCC have been registered over the past 20 years. Since multiple pathways are dysregulated in cancer, hence drugs targeting different pathways and their combinations may decrease the chances of cancer resistance, thereby improve outcomes. Other works are using novel therapeutic agents with different mechanisms of action, including telaglenastat (a glutaminase inhibitor), entinostat (HDAC inhibitor) and olaparib, talazoparib (poly (ADP-ribose) polymerase inhibitors) widely used in other tumors^33^. Eventually the goal of treatment, even in the metastatic setting, is cure and/or long-term survival. In most patients with advanced or mRCC who require systemic therapy, immune checkpoint inhibitor–based combination therapy is currently considered the frontline standard of care. Monotherapy with VEGF or mTOR inhibitors is no longer a reasonable option in the frontline setting except in unusual circumstances (e.g., immunotherapy ineligibility)^34^. Therefore, multiple efficient therapeutic strategies and agents are needed to improve the clinical outcome for treatment of mRCC. Development of novel anti-cancer compounds is often difficult, costly, time and resources intensive. Moreover, novel drugs may fail human trials due to unexpected lack of efficacy or safety problems. Therefore, repurposing of a drug is a promising alternative.

In the present study, we investigated the efficacy of Ketorolac against RCC. Ketorolac is a NSAID used to treat moderate to severe pain. It works by blocking COX1 and COX2, thereby decreasing production of prostaglandins^35^. Ketorolac is a racemate mixture of R and S and has been known to be active against several cancers^36^. Here we show for the first time the activity of Ketorolac against RCC by modulating tumor suppressor Par-4^37^. Our study evidently indicates significant anti-proliferative effect of Ketorolac in RCC and PDX cells alone as well as in combination with Sunitinib or Sorafenib in both 2D and 3D models. Interestingly, as per our observation the efficacy of Ketorolac alone was more pronounced in the 3D model than 2D assays. It is well known that 3D cell models more closely resemble the in vivo tumor with respect to tumor morphology, cell phenotypes and cell–cell interactions, tumor heterogeneity and the composition of tumor microenvironment (TME)^38^. Therefore, tumor cells’ response to anti-cancer drugs and drug penetration through the tumor tissue might be accurately captured in the 3D cultures. Given our observation of better efficacy in 3D model clearly indicates better diffusivity of Ketorolac through the 3D spheroids. Further, clonogenic assays which is widely accepted tool in tumor biology to measure tumor-initiating capacity and stem cell status was used to check the ability of Ketorolac to inhibit size, and number of colonies formed by cells alone and in combination with Sunitinib. Our data indicates strongly abolished clonogenic capacity of RCC cells. Importantly, Ketorolac showed significant downregulation of expression of proliferation and cell cycle markers like Cyclin D1, Ki-67 and DDX3 alone and in combination.

Rac-1 and Cdc42 are known to be over-expressed in several cancers and is associated with poor prognosis and drug resistance^39^. However, there is limited knowledge on the impact of Rac-1 and Cdc42 inhibition in RCC. We have shown that Ketorolac inhibits the transcript and protein level expression of Rac-1 and Cdc42 in A-498 cells alone as well as in combination with Sunitinib. Earlier studies have also demonstrated effect of Ketorolac in ovarian cancer through inhibition of Rac-1 and Cdc42^15^. Since, Rac-1 signaling has been known to promote angiogenesis via activation of HIF-1α, we looked at the ability of Ketorolac to modulate the expression pattern of HIF-1α. Our study showed that inhibition of Rac-1 and Cdc42 alone or in combination with Sunitinib profoundly inhibited the expression of *HIF-1α/DDX3/β-catenin* in A-498 xenograft model. Further *Tiam1*, a known oncogene is dysregulated in NSCLC, endometrial, colorectal, pancreatic, and cervical cancer and is known to activate both Rac-1 and Cdc42. Astoundingly, Ketorolac was also able to down regulate its transcript and protein level expression in A-498 cells. Notably, Ketorolac also caused down-regulation of *VEGF* at transcript and protein levels and significant upregulation of *E-cadherin* at the transcript levels indicative of its ability to prevent cellular migration and metastasis which is one of the major unmet need in RCC.

**Figure 8:**
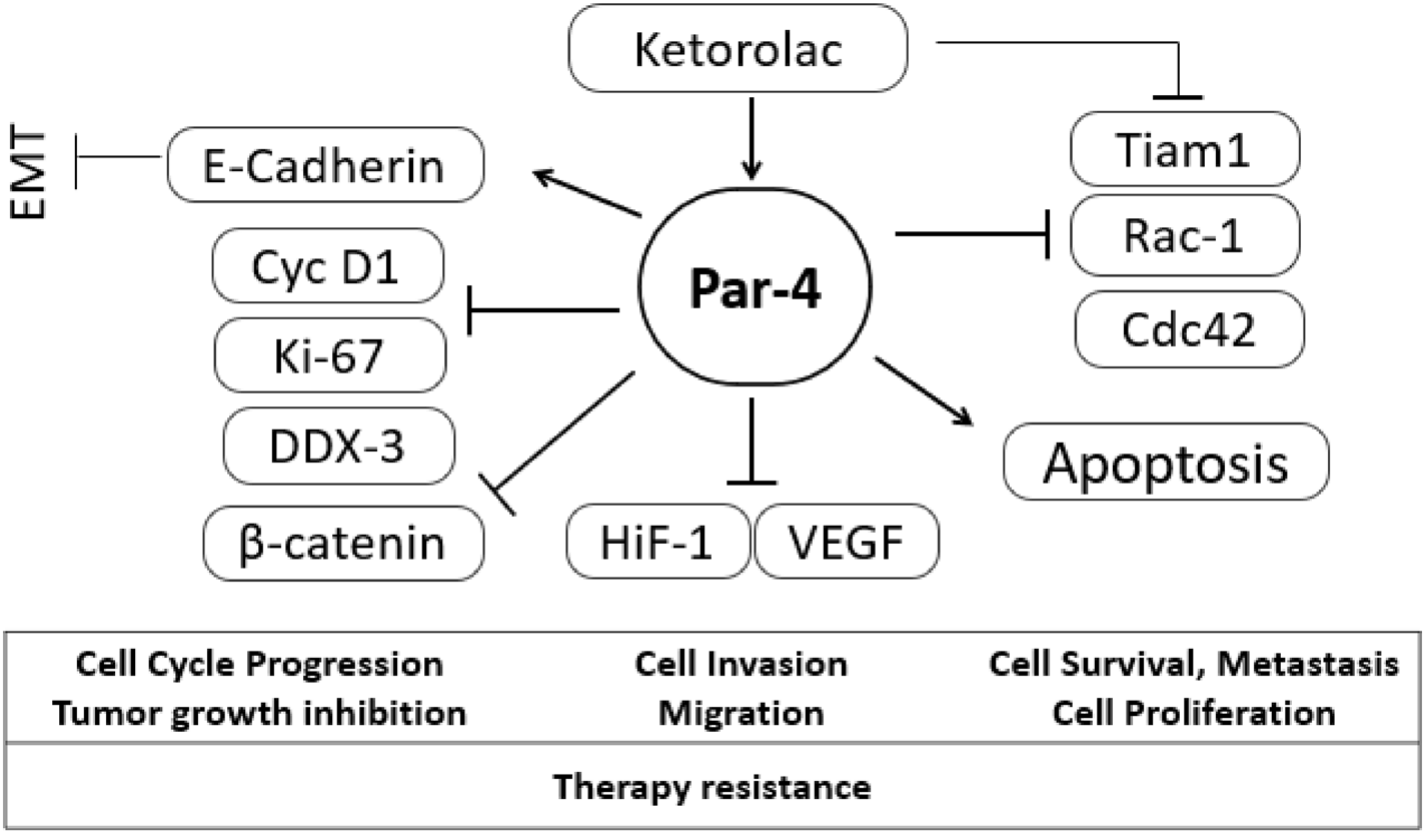
Proposed pathways targeted by Ketorolac in RCC.

Tumor suppressors are crucial factor in controlling cancer which play important roles in suppressing uncontrolled proliferation, immortality and tumorigenicity. Amongst several tumor suppressors, Par-4 is known to be ubiquitously expressed in different tissues across different species. Par-4 can selectively cause apoptosis in a wide variety of cancers cells, leaving normal cells unaffected. Our earlier studies have clearly shown the importance of Par-4 levels in glioblastoma and its applicability to be used as a prognostic marker in its treatment^31,40^. However, Par-4 is known to be down-regulated in over 70% of renal cancers, neuroblastoma, acute and chronic forms of leukemia^41^. It is therefore essential to restore or upregulate the levels of Par-4 in cancer as well as secretion through normal cells. Because the baseline levels of Par-4 secreted by normal cells are inadequate to cause massive apoptosis in cancer cells, secretogogues (small molecules that upregulates secretions) that upregulate the release of Par-4 by displacing Par-4 from its intracellular binding partners constitute an important therapeutic category^42^. Here, we have identified Ketorolac as a potent secretagogue which is able to upregulate the levels of Par-4 in both A-498 and normal mouse fibroblast, thereby indicating its ability to induce both autocrine and paracrine pathways. This mechanism of action of Ketorolac against RCC was confirmed using siRNA knockdown studies. Intriguingly, *Par-4* knockdown in A-498 cells led to a significant upregulation of *Rac-1* and *VEGF* while knockdown of *Rac-1* led to subsequent downregulation of *Cdc42* mediated by significant upregulation of *Par-4*. The data indicates that restoring Par-4 levels in cancer cells by using small molecule secretagogues like Ketorolac would not only lead to inhibition of angiogenic factors like HIF-1α and VEGF but also inhibition of cell growth, migration/invasion, polarity, adhesion, and cytoskeletal rearranging markers viz. Tiam1, Rac-1, Cdc42, DDX3, Cyclin D1 and β-catenin (Fig. 8). This appears to be the precise reason of the noteworthy in vivo anti-tumor activity of Ketorolac and its combination with Sunitinib, where 86% tumor regression was observed.

Thus, the present study indicates the competence of Ketorolac to regulate the expression of proliferation and metastasis pathways by its ability to upregulate Par-4 through autocrine and paracrine pathways. However, these astounding results obtained with A-498 cells further warrants studies using other RCC cell lines.

Furthermore, retrospective analyses have revealed that intraoperative administration of Ketorolac decreases the risk of breast cancer relapse^25,43^. Two institutional retrospective studies including 827 and 1007 patients evaluating the administration of Ketorolac show statistically significant association with a reduction of distant recurrences in patients with increased BMI. Recently, Panigrahy et al. have shown that preoperative, but not postoperative, administration of the nonsteroidal anti-inflammatory drug Ketorolac and/or Resolvins, eliminated micro-metastases in multiple tumor-resection models, resulting in long-term survival^22^. Ketorolac is currently available as a racemic mix of R and S enantiomer and the combined duration of its use of IV or IM dosing is not to exceed 5 days. This is mainly due to the toxicity concerns associated with S-enantiomer of Ketorolac. Interestingly, recently pre-clinical toxicity studies have shown that R-Ketorolac was well tolerated^15^. Hence, R-Ketorolac alone or in combination with standard of care may hold promise for future clinical use in RCC setting.

## Conclusions

Perhaps ours is the first report where we point towards a specific mechanism of action of Ketorolac as a Par-4 up regulator both in the cancer as well as normal cells, thereby, suggesting that repurposing Ketorolac in conjunction with standard of care may serve as an effective therapeutic agent against RCC especially in metastatic setting.

## Supplementary Information

Supplementary Information 1.

Supplementary Information 2.

Supplementary Information 3.

## Data Availability

The datasets analyzed during the current study are available from the corresponding author upon request.
