# Supplementary material for: Ketorolac modulates Rac-1/HIF-1α/DDX3/β-catenin signalling via a tumor suppressor prostate apoptosis response-4 (Par-4) in renal cell carcinoma
Source: Sci Rep. 2023 Apr 6;13:5659. doi: 10.1038/s41598-023-32627-z (PMC10079967; doi:10.1038/s41598-023-32627-z)

**Additional File 1**

**Ketorolac modulates Rac-1/HIF-1α/DDX3/β-catenin signalling via a tumor suppressor Prostate apoptosis response-4 (Par-4) in renal cell carcinoma**

**Authors**

Vinay Sonawane^1†^, Jeevan Ghosalkar^1†^, Swati Achrekar^1^, Kalpana Joshi^1*^

**Affiliations**

^1^Cell Biology Division, Cipla Ltd., Vikhroli West, Mumbai - 400083, INDIA.

***Correspondence author**

Kalpana Joshi, Cell Biology Division, Cipla Ltd, LBS Marg, Vikhroli West, Mumbai – 400083, INDIA, Tel: +91-22-25766326, Email: [kalpana.joshi@cipla.com](mailto:kalpana.joshi@cipla.com)

**^†^**Contributed equally to this work

This additional file contains (Table 1, 2, Fig 3 and Fig 4)

**Table 1: Tumor models tested in the clonogenic assay**

| Tumor Designation | Tumor Number | Stage at implantation | Histology | Differentiation | Patient Age at Surgery | Gender | Material used for study |
| --- | --- | --- | --- | --- | --- | --- | --- |
| RFX | 1183 | NA | Clear cell carcinoma | Not known | 74 | Male | PDX Suspension |
| RFX | 1220 | T3M1 lung | Clear cell carcinoma | Not known | 59 | Male | PDX Suspension |
| RFX | 1781 | Recurrence | Clear cell carcinoma | Poor | 43 | Male | PDX Suspension |
| RFX | 393 | T2NxM1 (lung) | Clear cell carcinoma | Poor | 54 | Male | PDX Suspension |
| RFX | 486 | T3N0M1 lung GII | Hypernephroma | Not known | 51 | Female | Cell line (NCI) |
| RFX | 786-O | NA | Adenocarcinoma | Not known | 58 | Male | Cell line (NCI) |
| RFX | A-498 | NA | Carcinoma | Not known | 52 | Female | Cell line (ECACC) |
| RFX | ACHN | NA |  | Not known | 22 | Male | Cell line (NCI) |
| RFX | Caki-1 | NA | Clear cell carcinoma | Not known | 49 | Male | Cell line (NCI) |
| RFX | SN12C | NA | Carcinoma |  |  | NA | Cell line (NCI) |

Table 1: Represents histology in panel of RCC cell lines along with IC_50_ values for Ketorolac in 2D and 3D assay and for Sunitinib in 2D assay

| **PDX** | **Model** | **Histology** | **Age**  **/Gender** | **Differentiation** | **Ketorolac** | | | | **Sunitinib** | **Sorafenib** |
| --- | --- | --- | --- | --- | --- | --- | --- | --- | --- | --- |
|  |  |  |  |  | **IC_50_ - 2D (mM)** | **IC_50_ -3D**  **(mM)** | **IC_50_ - 3D**  **(µM)** | | | **IC_50_ - 3D**  **(µM)** |
| RXF | 1183L | ccRCC | 74/M | Not known | 4.12 | 1.55 | 1.038 | | | 8.10 |
| RXF | 1220L | ccRCC | 59/M | Not known | 9.02 | 1.76 | 0.78 | | | NA |
| RXF | 1781L | ccRCC | 43/M | Poor | 6.22 | 1.90 | 0.56 | | | NA |
| RXF | 393L | ccRCC | 54/M | Not known | 4.12 | 2.83 | 0.43 | | | NA |
| RXF | 486L | Hypernephromas | 51/F | Not known | 6.87 | 2.68 | 39.97 | | | 8.15 |
| **CDX** | **Model** |  |  |  |  |  | | |  |  |
| RXF | 786-O | Adenocarcinoma | 58/M | Not known | 3.25 | 3.80 | | 1.97 | | 11.62 |
| RXF | A-498 | Carcinoma | 52/F | Not known | 6.35 | 0.28 | | 1.61 | | 7.6 |
| RXF | ACHN | pRCC | 22/M | Not known | 5.47 | 3.14 | | 3.39 | | NA |
| RXF | Caki-1 | ccRCC | 49/M | Not known | 2.80 | 3.68 | | 4.25 | | NA |
| RXF | SN12C | Carcinoma | NA | Not known | 4.35 | 2.60 | | 2.87 | | 13.69 |

**Table 2: Histology of RCC tumor cell lines**

| Sr No | Gene | Primer sequence |
| --- | --- | --- |
| 1 | *Par-4_F.P* | 5' GCCGCAGAGTGCTTAGATGAG 3' |
|  | *Par-4_R.P* | 5' GCAGATAGGAACTGCCTGGATC 3' |
| 2 | *Tiam1_F.P* | 5' CTCGTCAGGGGGTGTACGAG 3' |
|  | *Tiam1_R.P* | 5' ACCGGTGCATTTGGCACATAGCCG 3' |
| 3 | *Rac-1_F.P* | 5' GCGTTGCCATTGAACTCACC 3' |
|  | *Rac-1_R.P* | 5' GAGCTGCTACGCTCACTCCATTAC 3' |
| 4 | *Cdc 42_F.P* | 5' ACATCTGTTTGTGGATAACTCA 3' |
|  | *Cdc42_R.P* | 5' GGGAGCCATATACTCTTGGA 3' |
| 5 | *Cyclin D1_F.P* | 5´ GGCGGAGGAGAACAAACAGA 3´ |
|  | *Cyclin D1_R.P* | 5' TGTGAGGCGGTAGTAGGACA 3' |
| 6 | *DDX3- F* | 5' GGAGGAAGTACAGCCAGCAAAG 3' |
|  | *DDX3 - R* | 5' CTGCCAATGCCATCGTAATCACTC 3' |
| 7 | *KI-67 - F* | 5' CGACCCTACAGAGTGCTCAACAAC 3' |
|  | *KI-67 - R* | 5' AACTGCGGTTGCTCCTTCACT 3' |
| 8 | *VEGFA_FP* | 5' CTGGAGCGTGTACGTTG 3' |
|  | *VEGFA_RP* | 5'TTTAACTCAAGCTGCCTCGC 3' |
| 9 | *Hif- 1 alpha_F.P* | 5´ TTCACCTGAGCCTAATAGTCC 3´ |
|  | *Hif 1 alpha_R.P* | 5´CAAGTCTAAATCTGTGTCCTG 3´ |
| 10 | *Gapdh F.P.* | 5' cgagatccctccaaaatcaa 3' |
|  | *Gapdh_R.P* | 5' atccacagtcttctgggtgg 3' |
| 11 | *E Cadherin-1_F.P* | 5' ATGGCTGAAGGTGACAGAGC 3' |
|  | *E Cadherin-1_R.P* | 5' CACCTTCCATGACAGACCCC 3' |

**Figure 1:** Primer sequences used for gene expression

**Figure 2: %** Mean Body weight Change for Mice bearing A-498 tumor


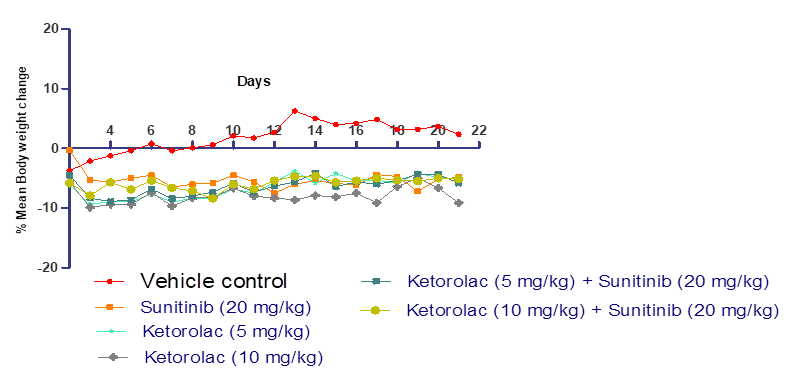

Supplement: Supplementary file 1 — Supplementary Information 1. [file 41598_2023_32627_MOESM1_ESM.docx]
