# Supplementary material for: Ketorolac modulates Rac-1/HIF-1α/DDX3/β-catenin signalling via a tumor suppressor prostate apoptosis response-4 (Par-4) in renal cell carcinoma
Source: Sci Rep. 2023 Apr 6;13:5659. doi: 10.1038/s41598-023-32627-z (PMC10079967; doi:10.1038/s41598-023-32627-z)
Supplement: Supplementary file 2 — Supplementary Information 2. [file 41598_2023_32627_MOESM2_ESM.docx]

**Additional File 2 for**

**Ketorolac modulates Rac-1/HIF-1α/DDX3/β-catenin signalling via a tumor suppressor Prostate apoptosis response-4 (Par-4) in renal cell carcinoma**

**Authors**

Vinay Sonawane^1†^, Jeevan Ghosalkar^1†^, Swati Achrekar^1^, Kalpana Joshi^1*^

**Affiliations**

^1^Cell Biology Division, Cipla Ltd., Vikhroli West, Mumbai - 400083, INDIA.

***Correspondence author**

Kalpana Joshi, Cell Biology Division, Cipla Ltd, LBS Marg, Vikhroli West, Mumbai – 400083, INDIA, Tel: +91-22-25766326, Email: [kalpana.joshi@cipla.com](mailto:kalpana.joshi@cipla.com)

**^†^**Contributed equally to this work

This additional file contains Fig 1 to Fig 9

**Figure 1:** Raw data of VEGF immunoblot from Figure 3H

**48 h**


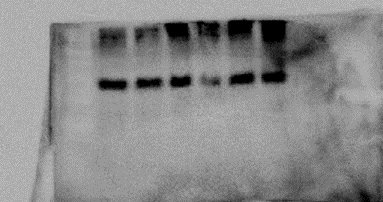


*VEGF*

*20 KDa*

**24 h**

**
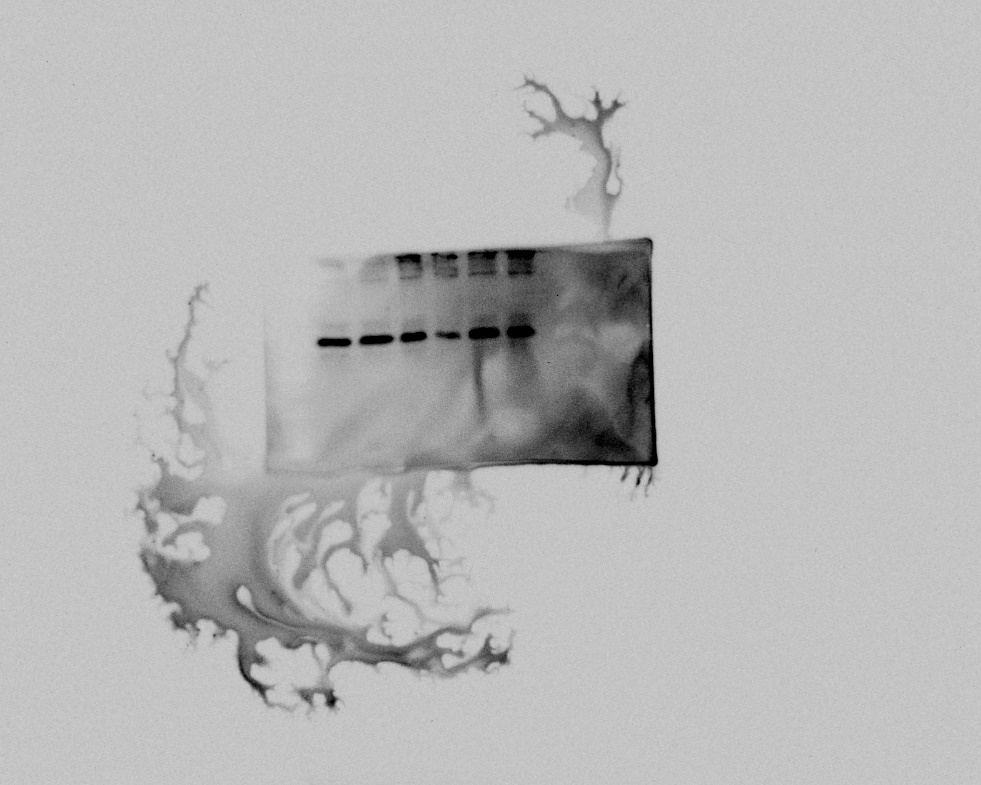
**

*VEGF*

*20 kDA*


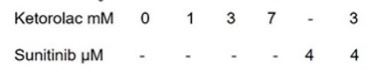


GAPDH

36 bp


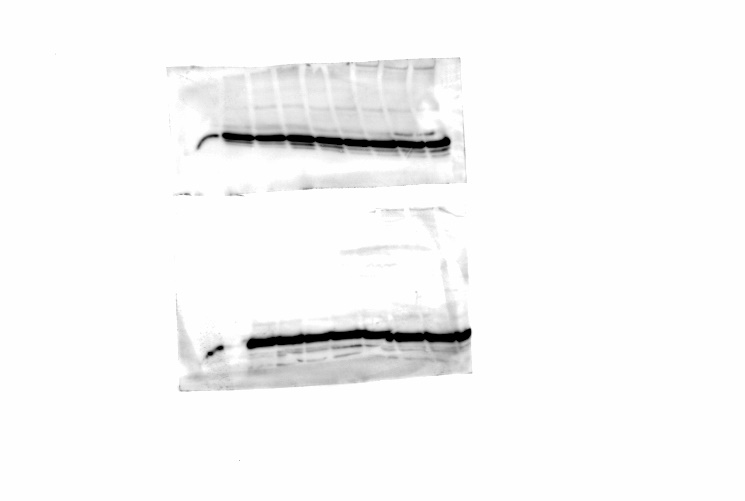

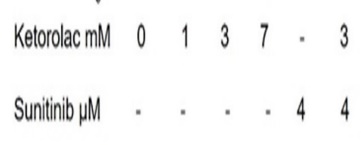


GAPDH

36 bp


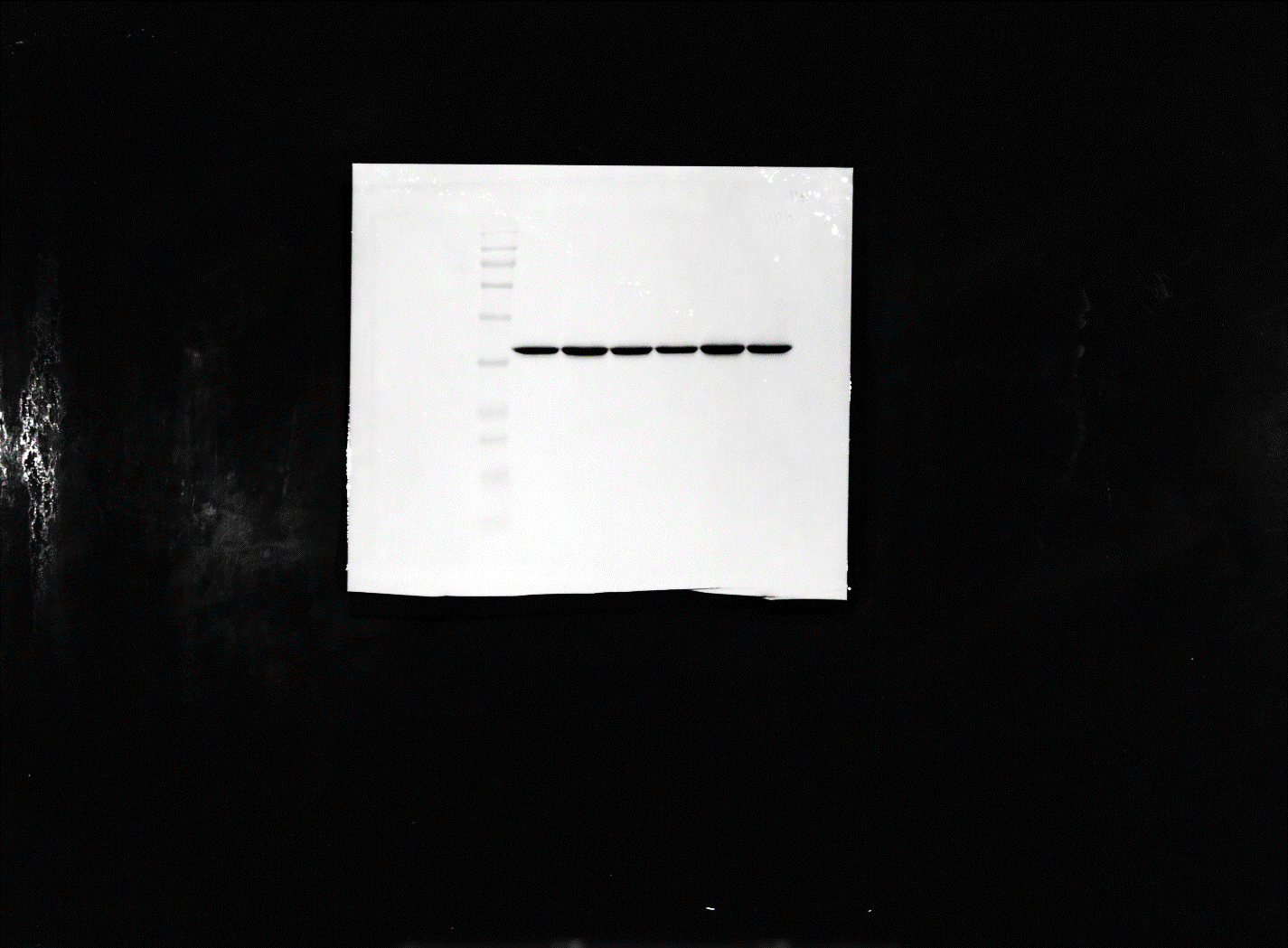


**Figure 1:** Raw data of VEGF immunoblot from Figure 3H


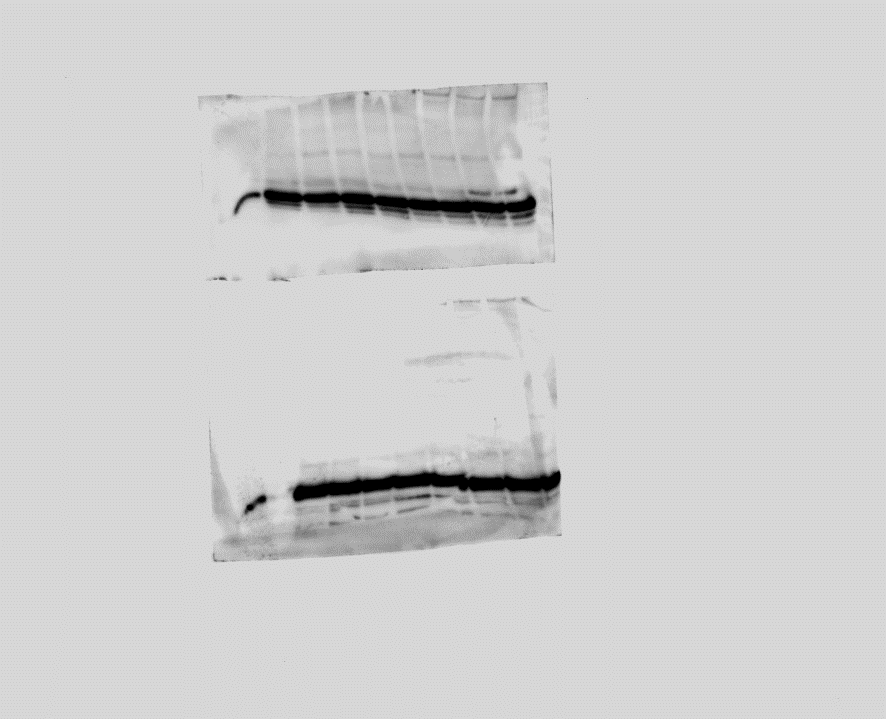


**GAPDH**

**36 bp**


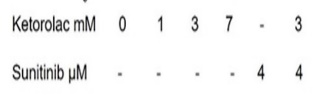


**72 h**


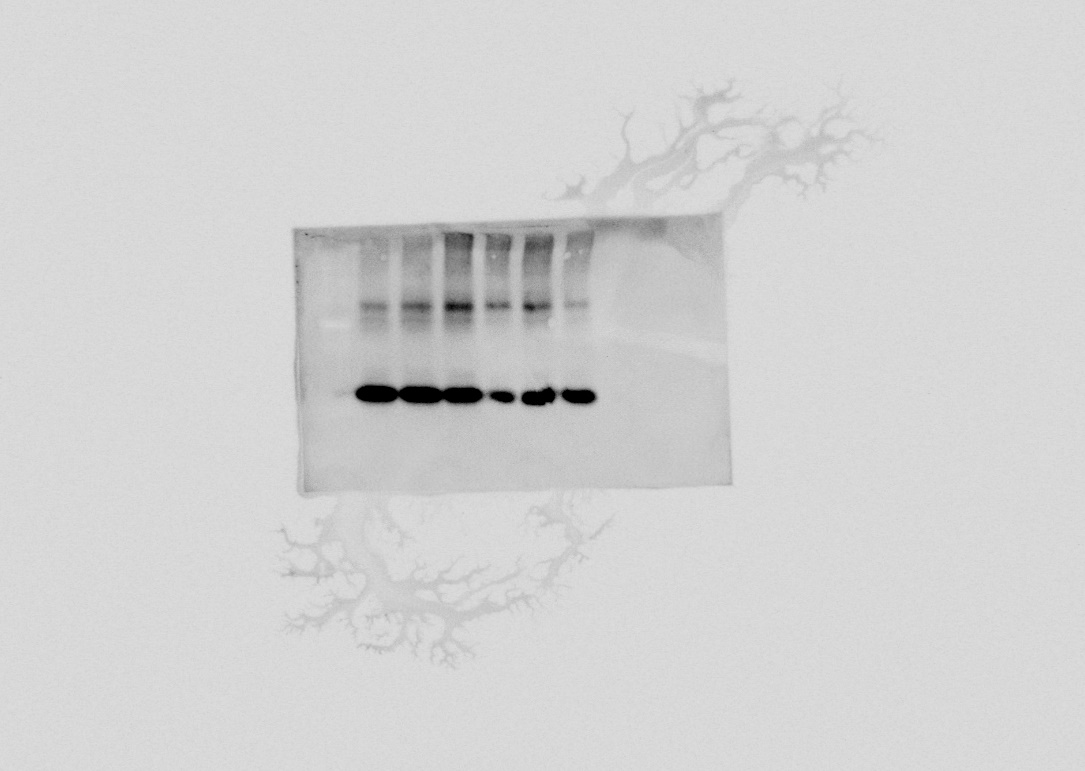


**VEGF**

**20 bp**

**Figure 2:** Raw data of cyclin D1, Ki-67, GAPDH immunoblot from Figure 4C


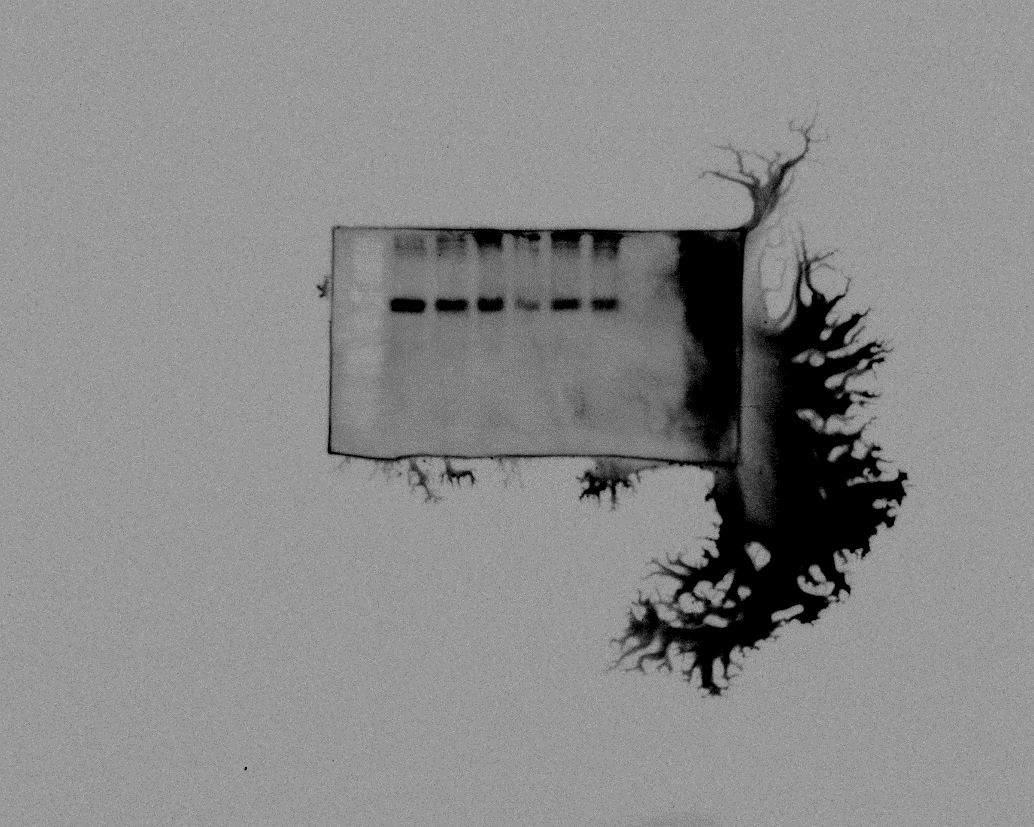


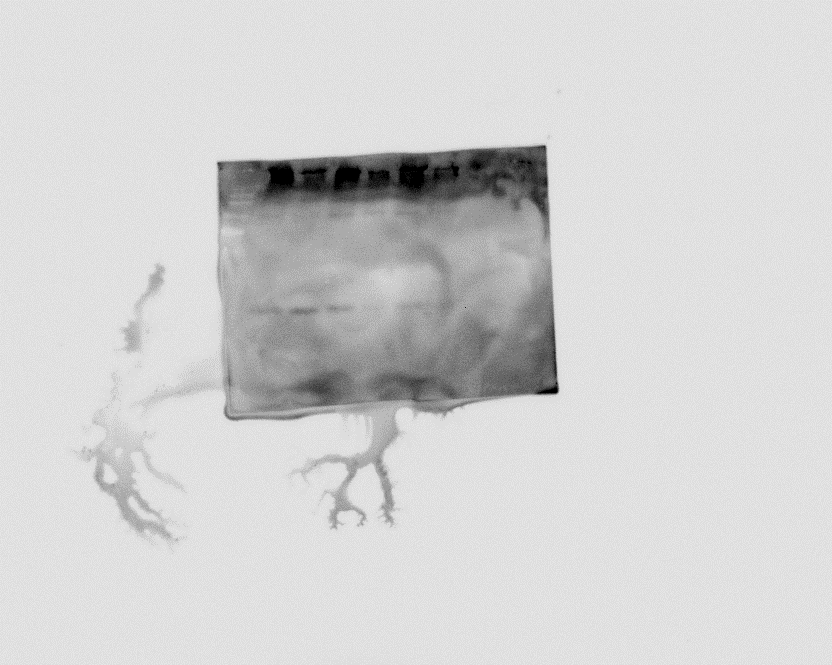


**Ki-67**

**(359 kDa)**

**Cyclin D1**

**36 KDa**


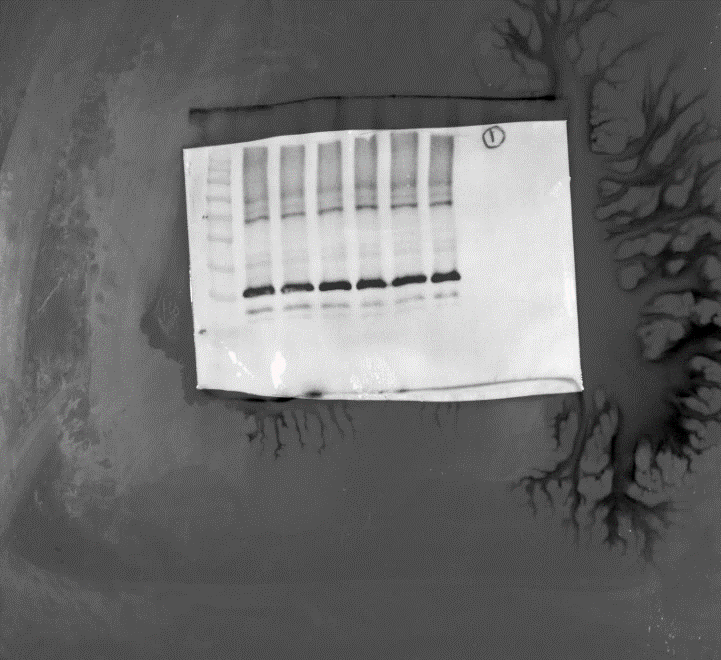

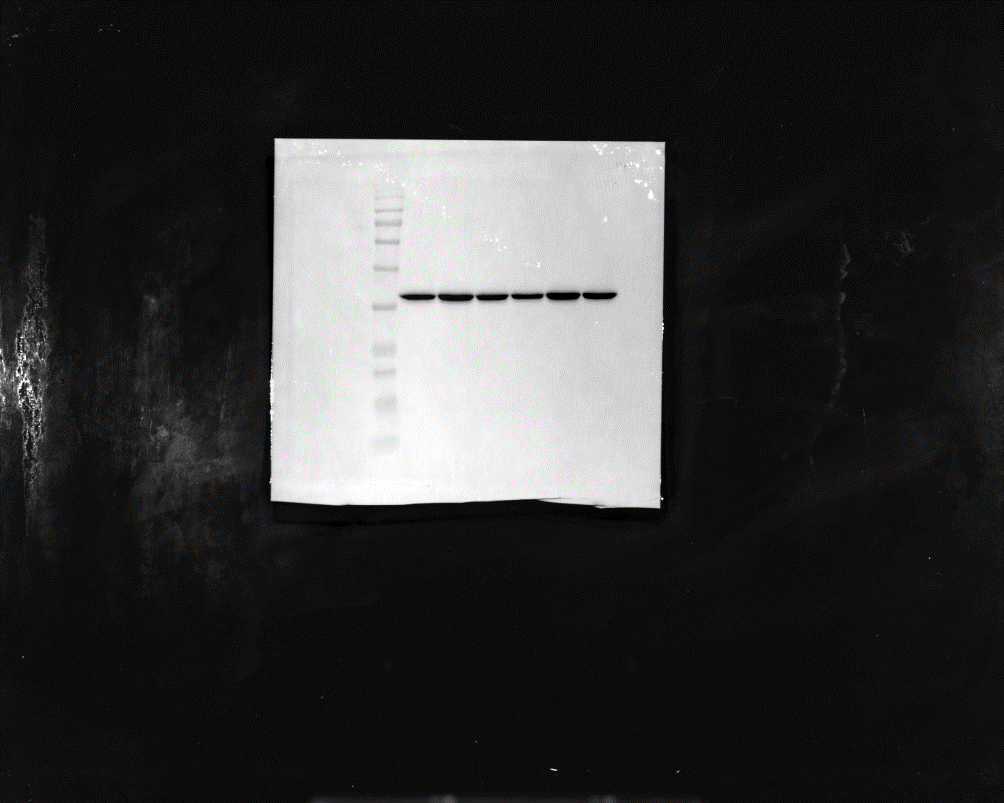


**GAPDH**

**(36 kDa)**

**GAPDH**

**(36 kDa)**

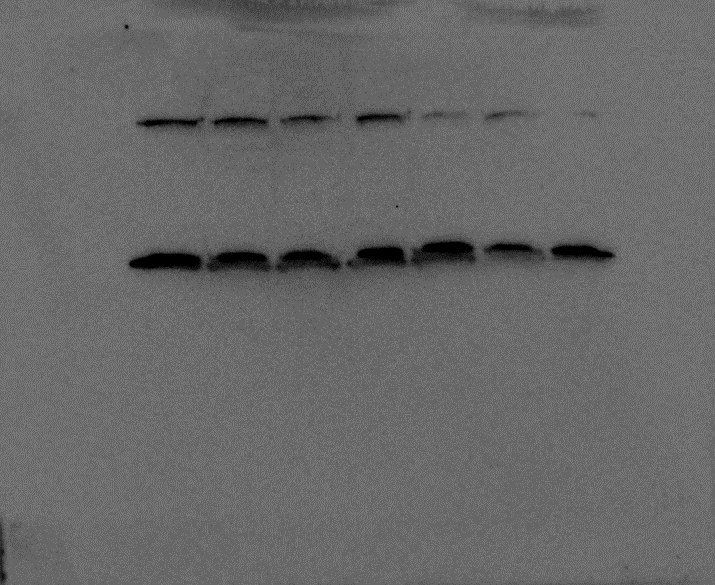


**DDX-3**

**73 KDa)**


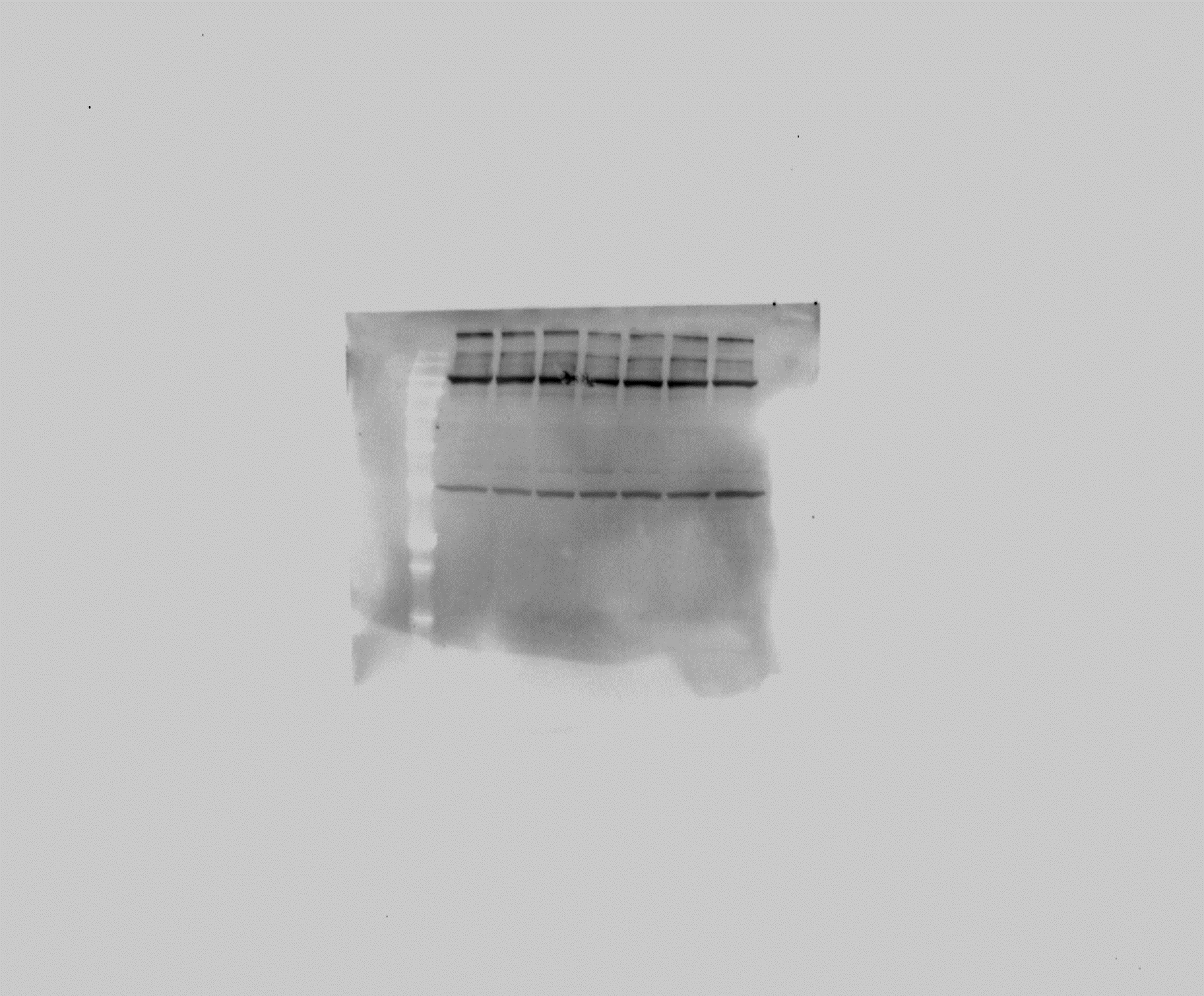
**Figure 2:** Raw data of cyclin D1, Ki-67, GAPDH immunoblot from Figure 4C

**pRB**

**105 KDa)**

**Figure 3:** Raw data for agarose gel of Figure 5A


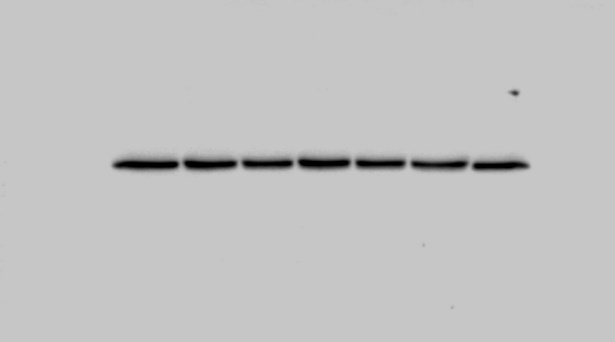


**GAPDH**

**36 KDa)**


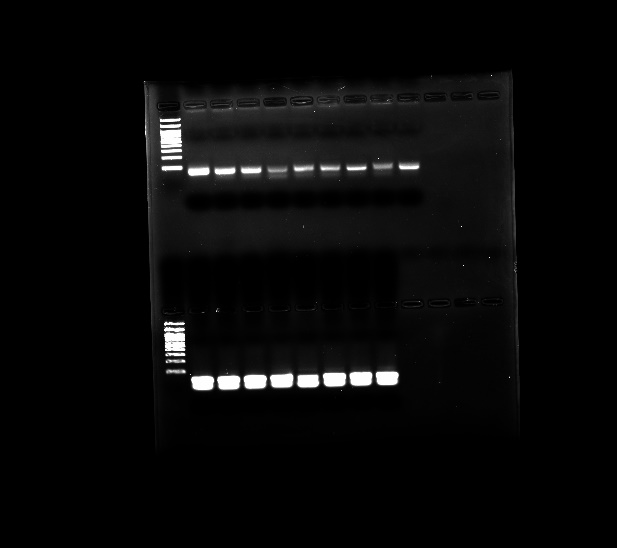


*Par-4*

*137 bp*


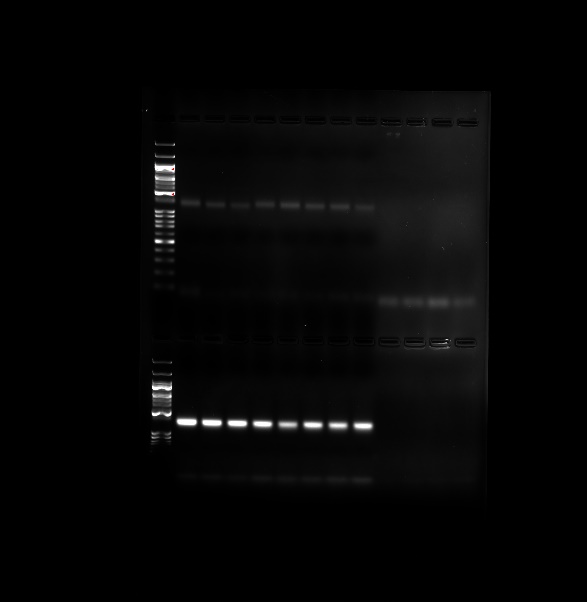


**Figure 3:** Raw data for agarose gel of Figure 5B

*Gapdh*

*234 bp*


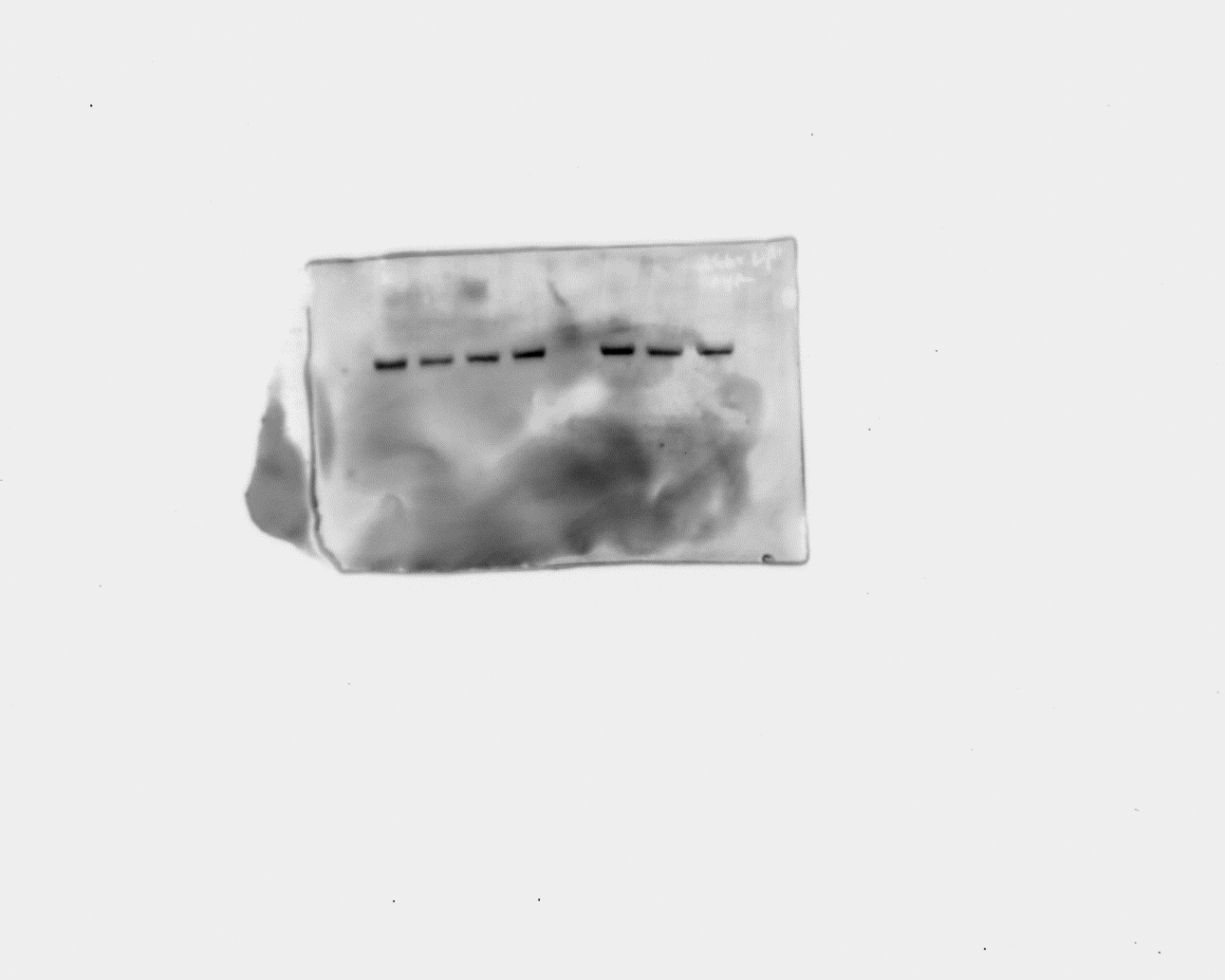


**
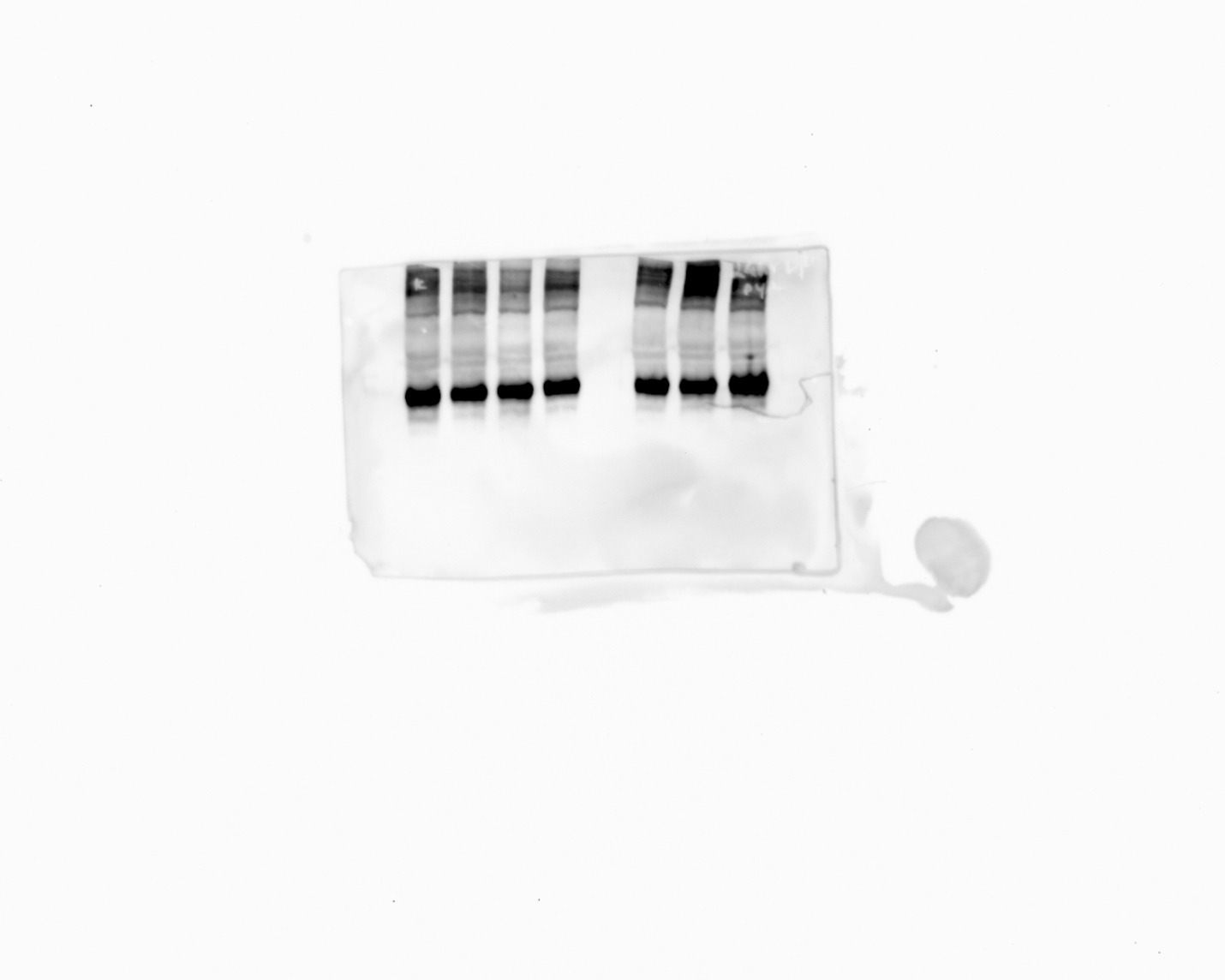
**

GAPDH

36 kDA

Par-4

36 kDA

**
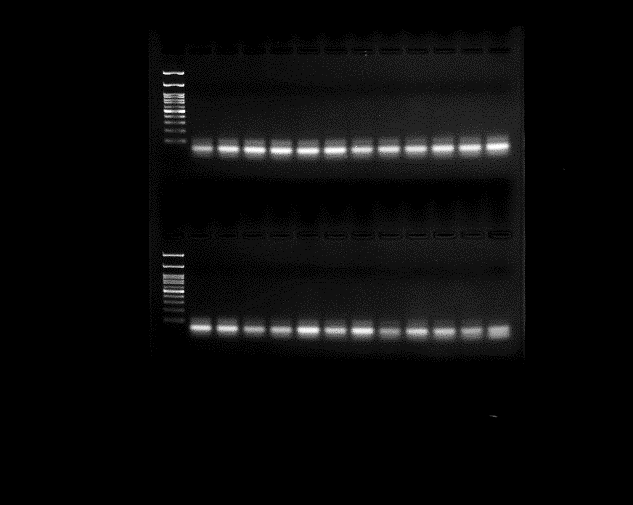
Figure 4:** Raw data for agarose gel of Figure 5C and 5D

**
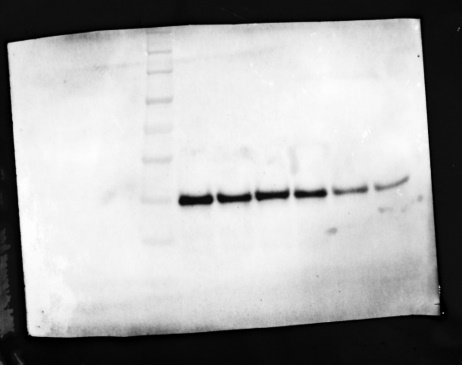
**

*Par-4*

*(137 bp)*

Par-4

(36 kDa)


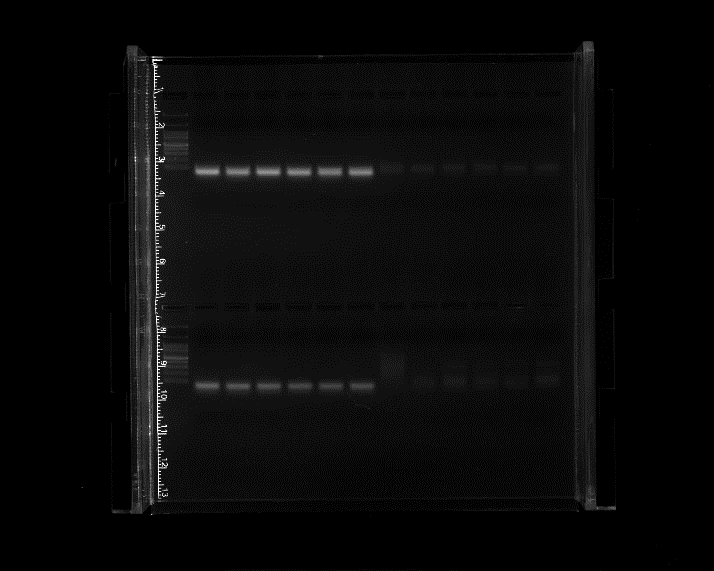

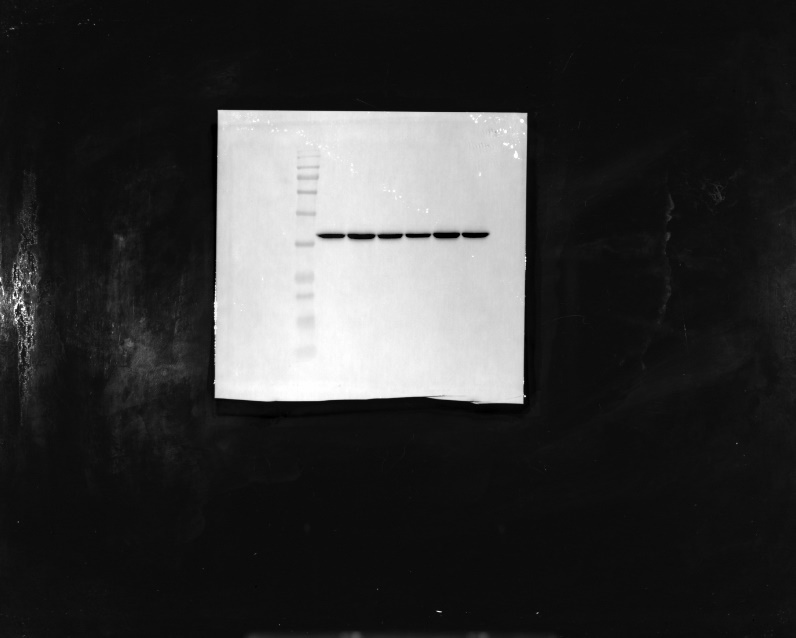


*gapdh*

(234 bp)

GAPDH

(36 kDa)


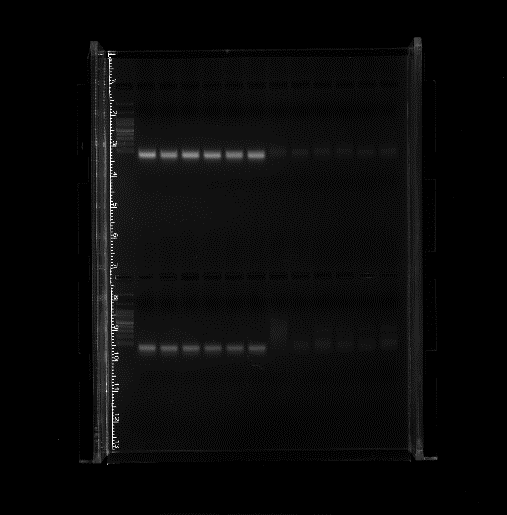

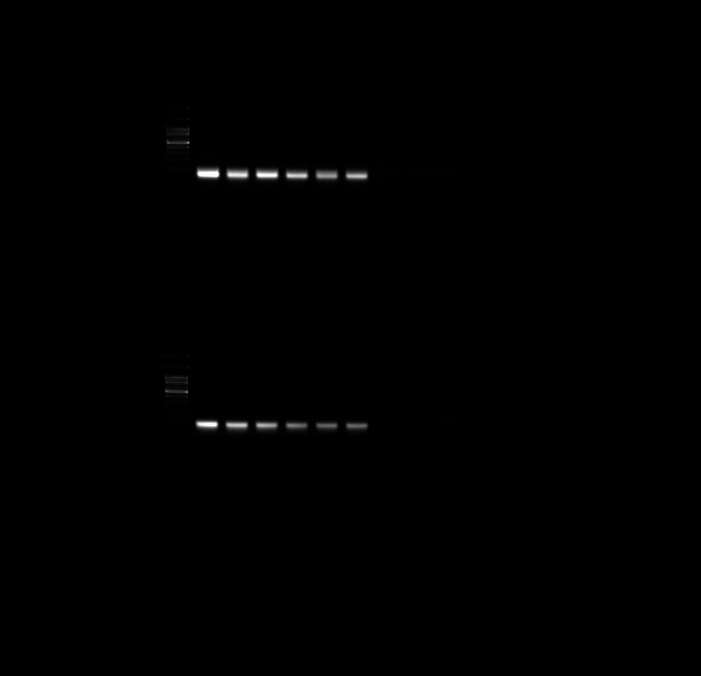

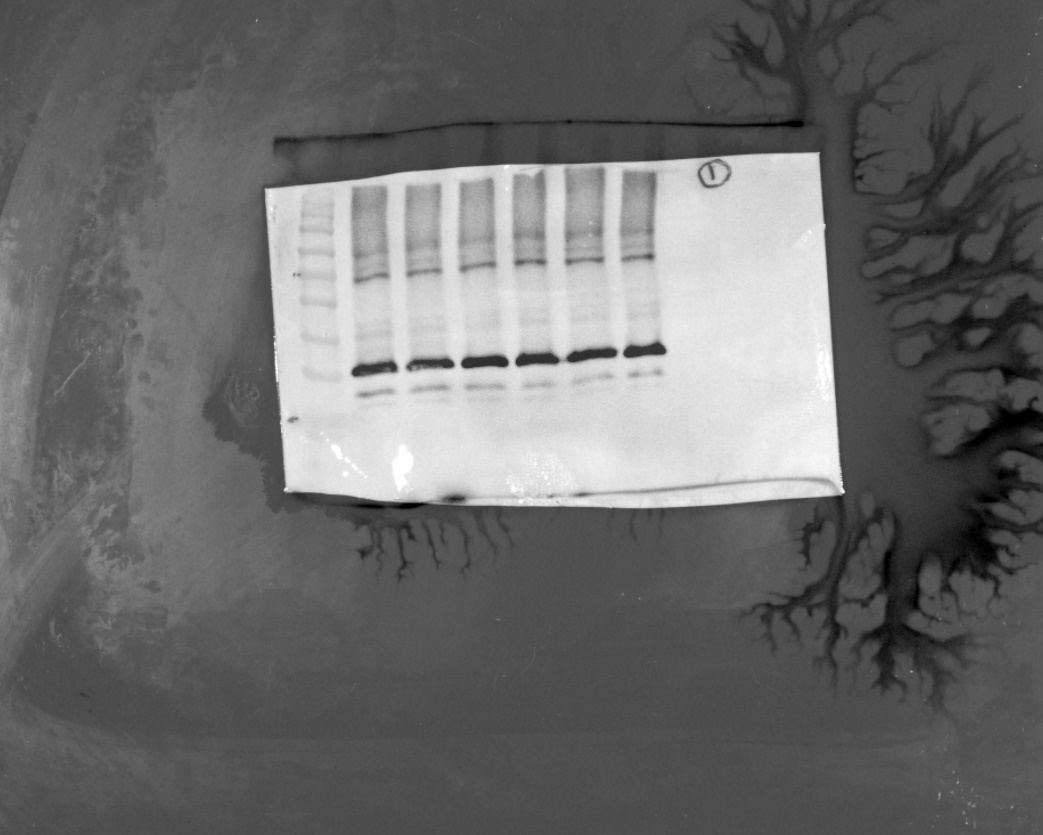

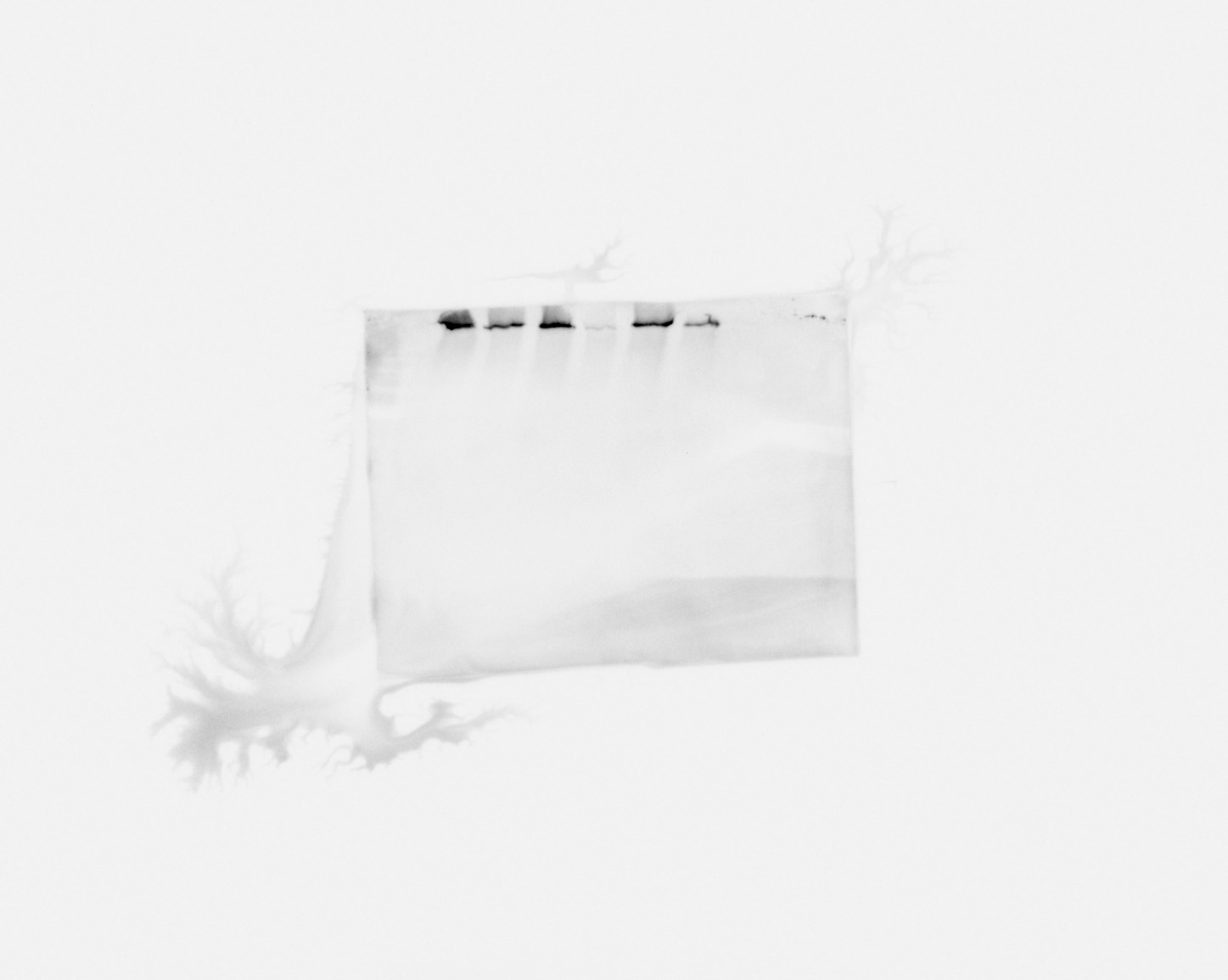
**Figure 4:** Raw data for agarose gel of Figure 5C and 5D

*Tiam1*

(318 bp)

GAPDH

(36 kDa)

Tiam1

(177 KDA)

*gapdh*

(234 bp)

**
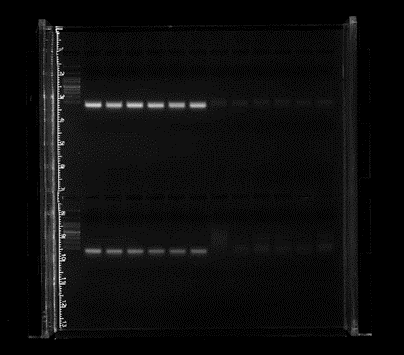
Figure 4:** Raw data for agarose gel of Figure 5C and 5D

*gapdh*

(234 bp)


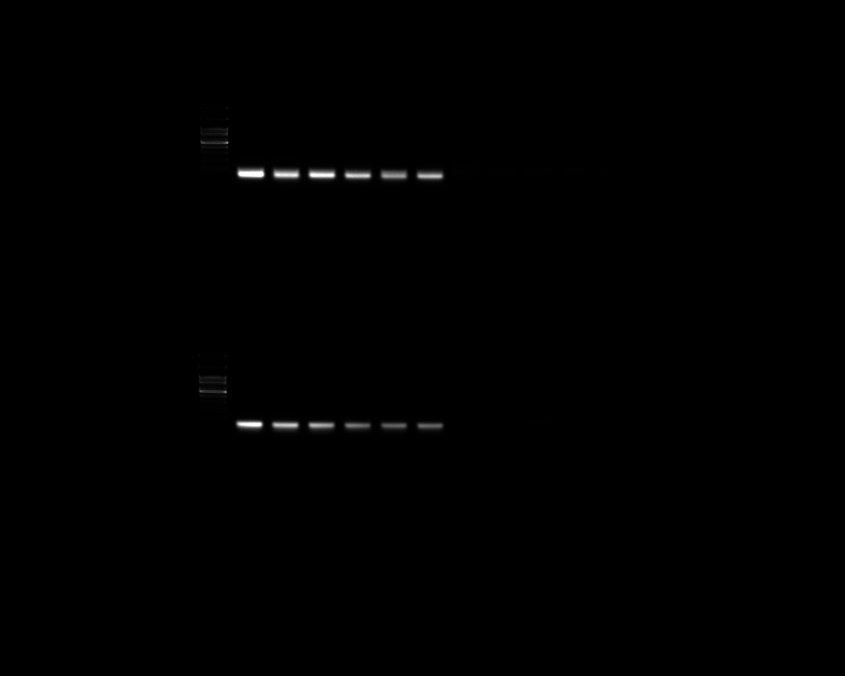

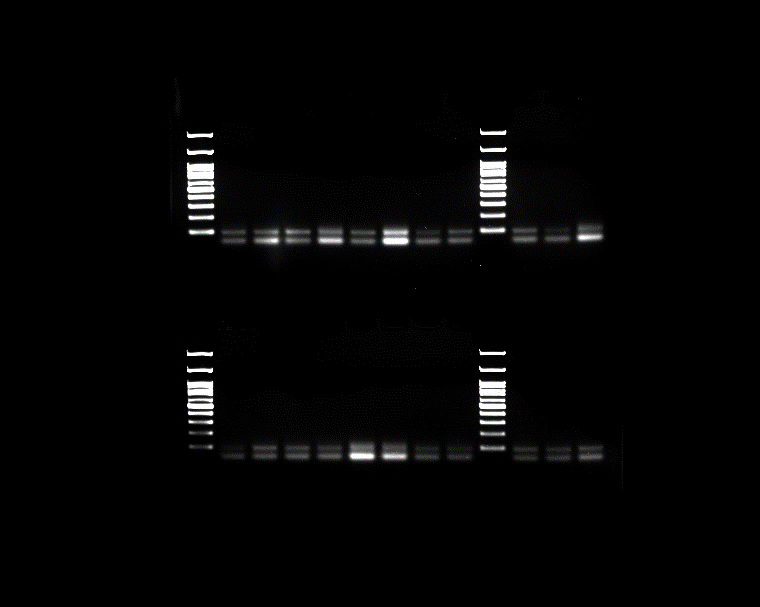

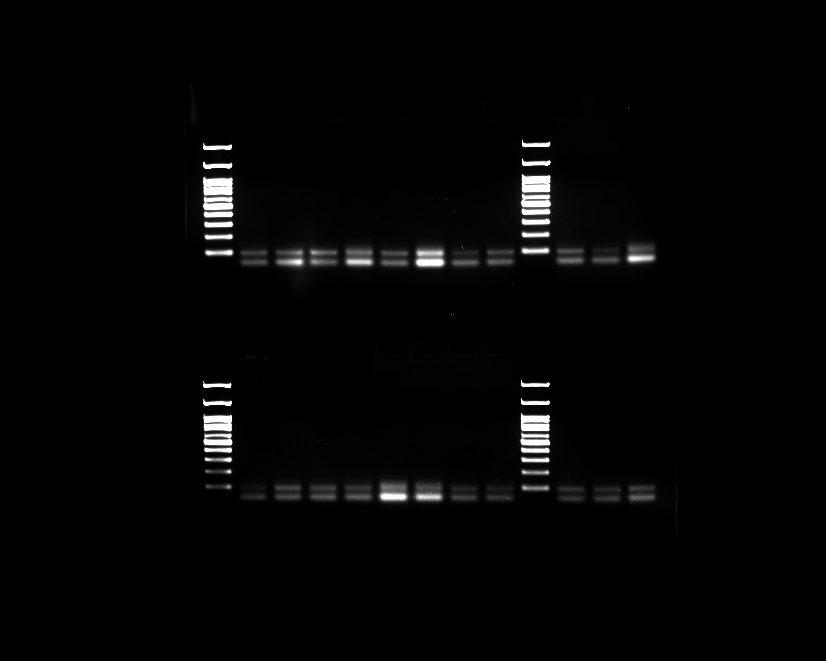
**Figure 5:** Raw data for agarose gel of Figure 6A

*Cdc42*

(101 bp)


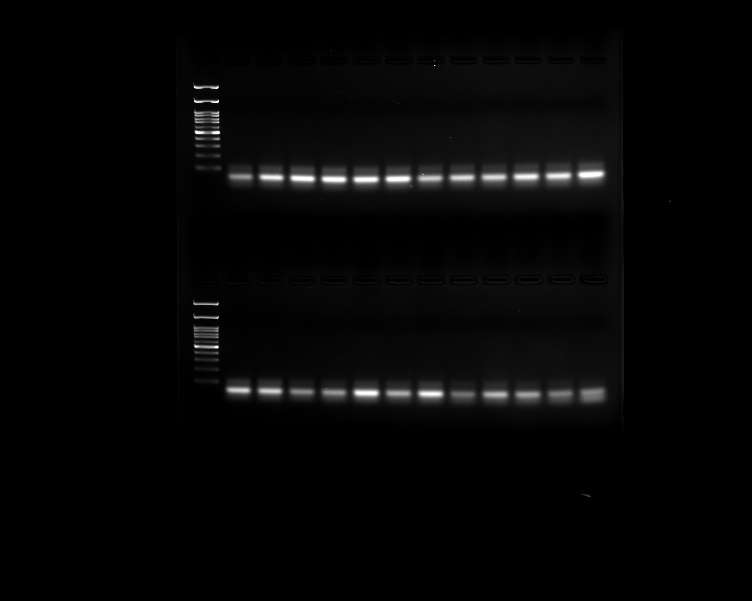


*Rac-1*

(91 bp)


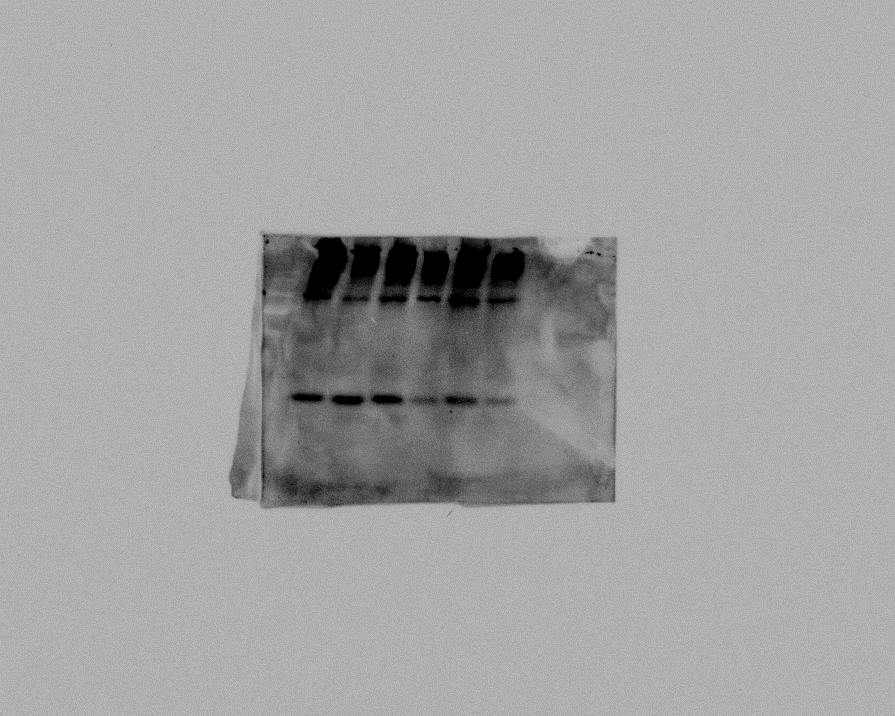


Rac1

(21kDa)


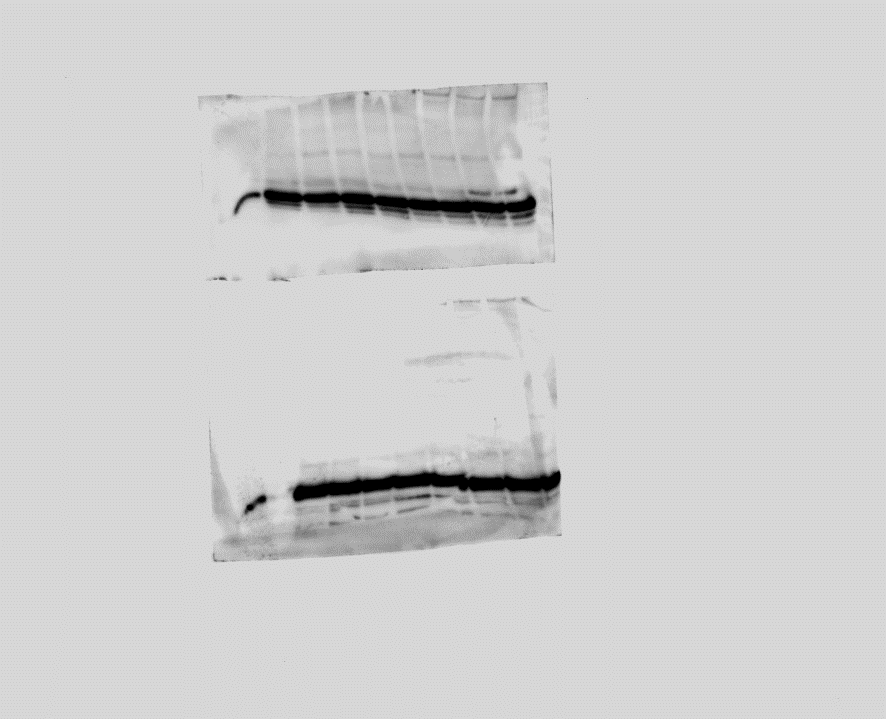


GAPDH

(36 kDa)


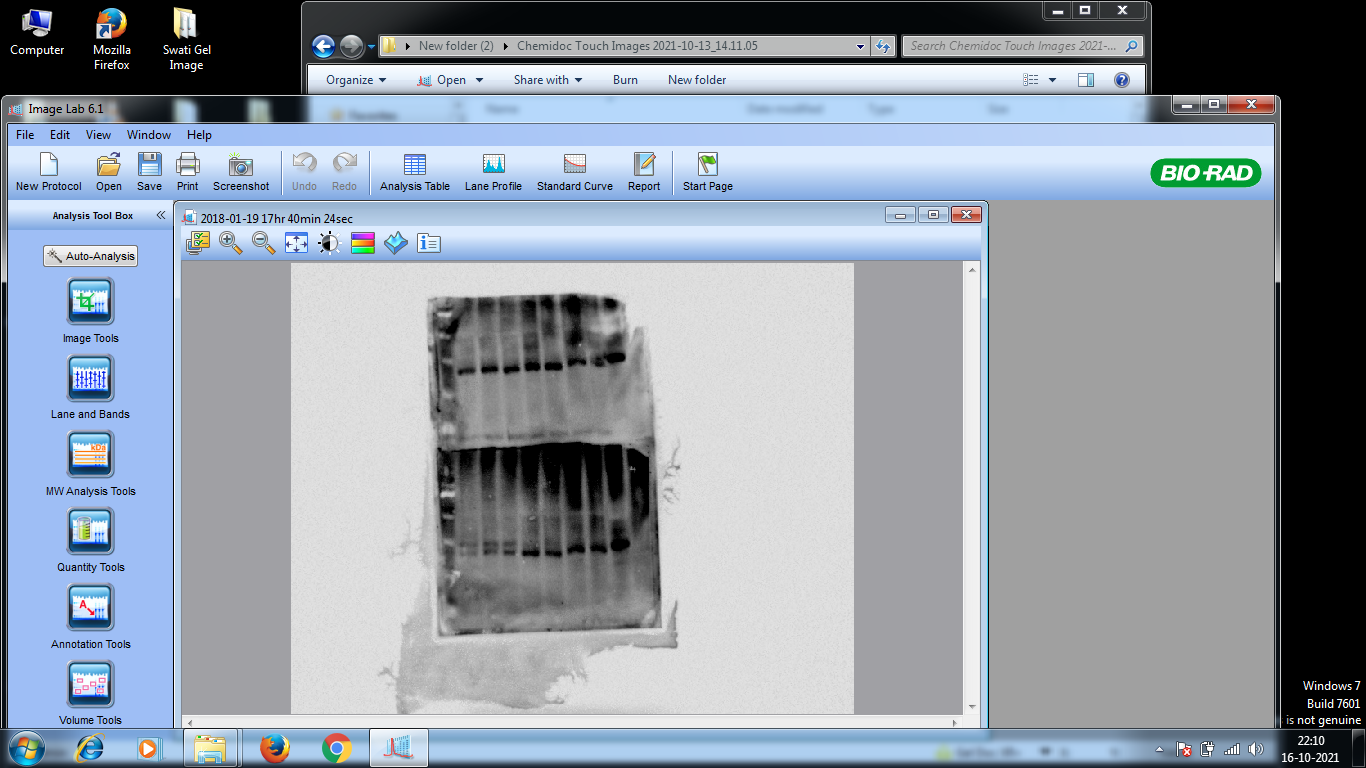


Cdc42

(21kDa)

***Par-4***

**137 bp**

24h

2h

5h


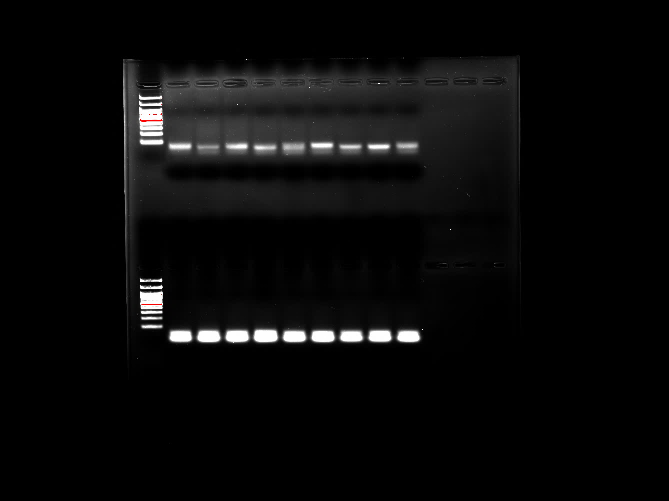


18h

5h

18h


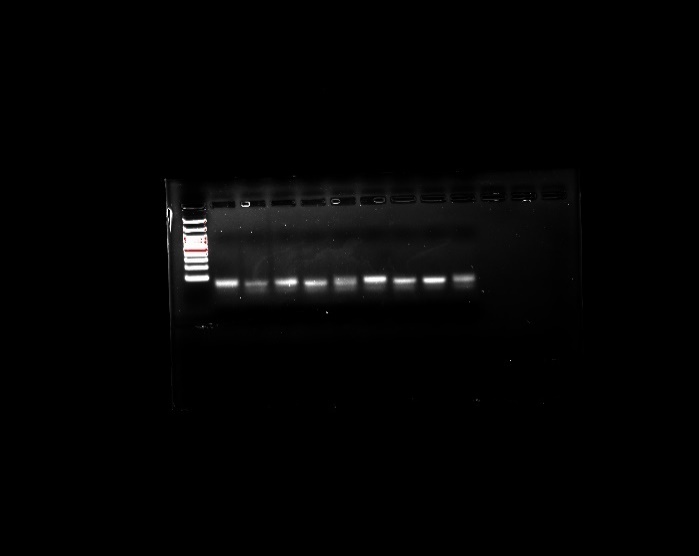


2h

***Rac-1***

***84 bp***

24h

24h

24h

**
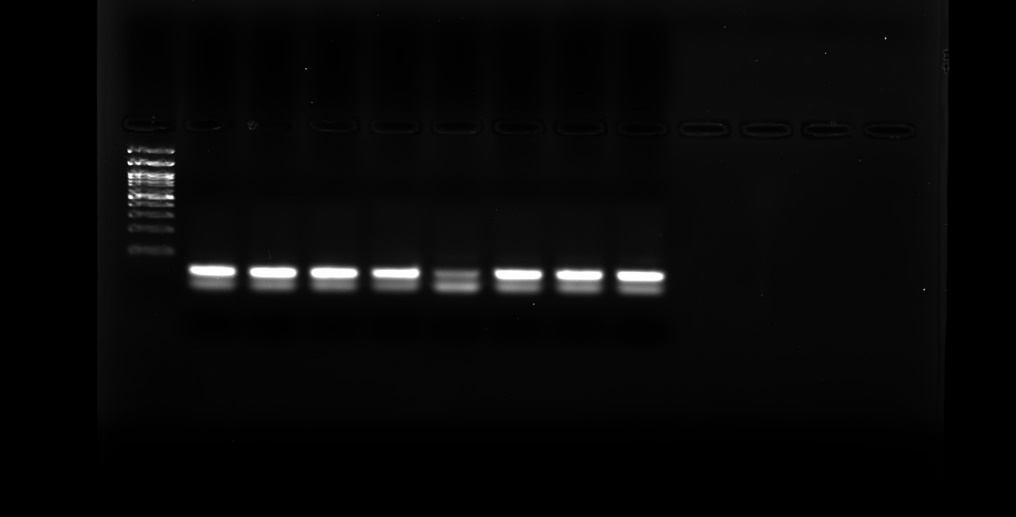
**
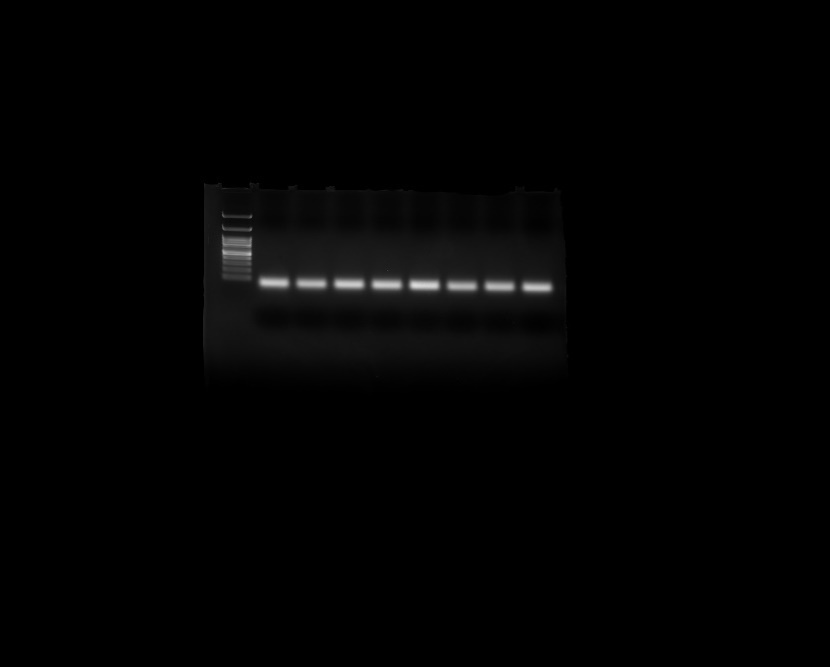


5h

2h

18h

24h

***gapdh***

**227 bp**

**Figure 6:** Raw data for agarose gel of Figure 6B


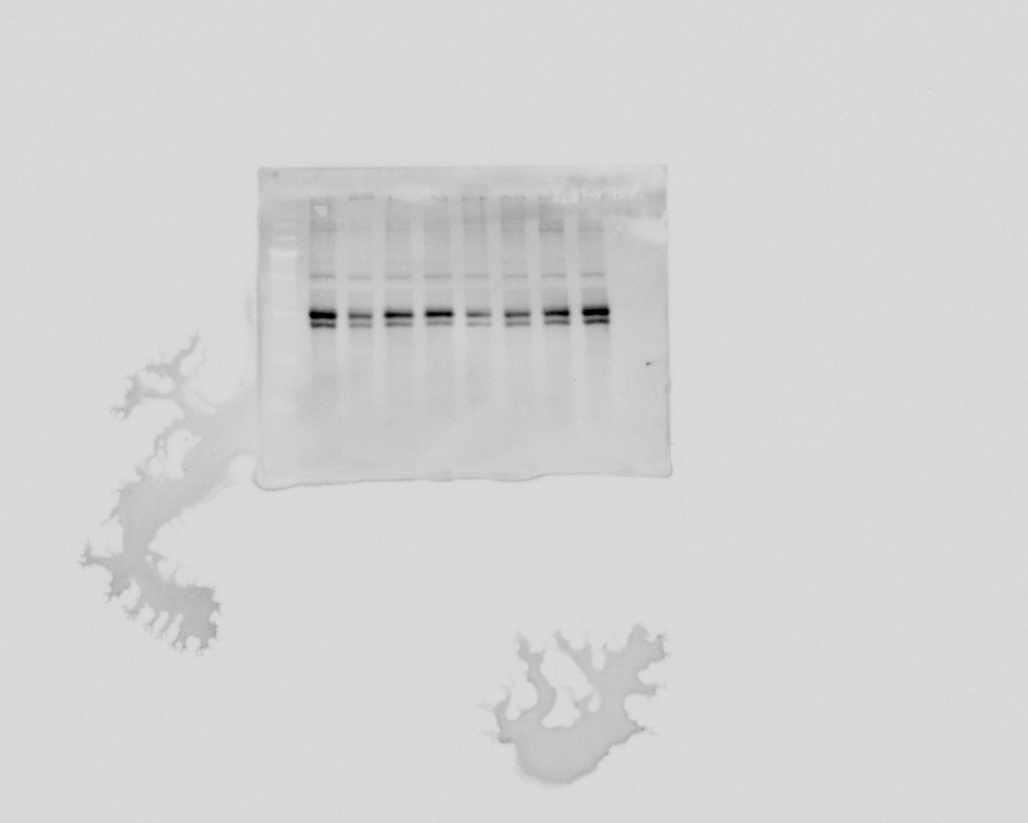


**Par-4**

**36 kDa**


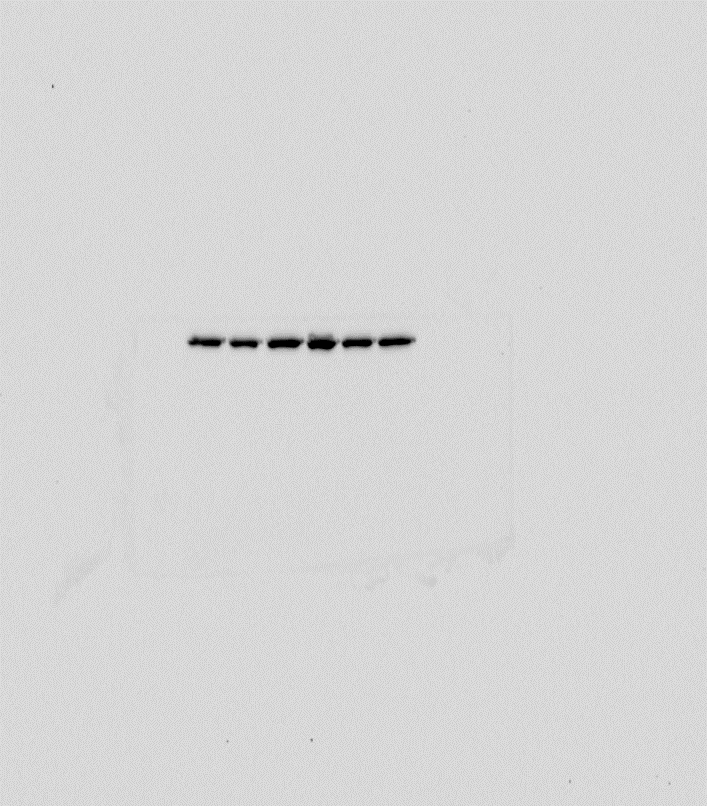


**Rac-1**

**20 kDa**


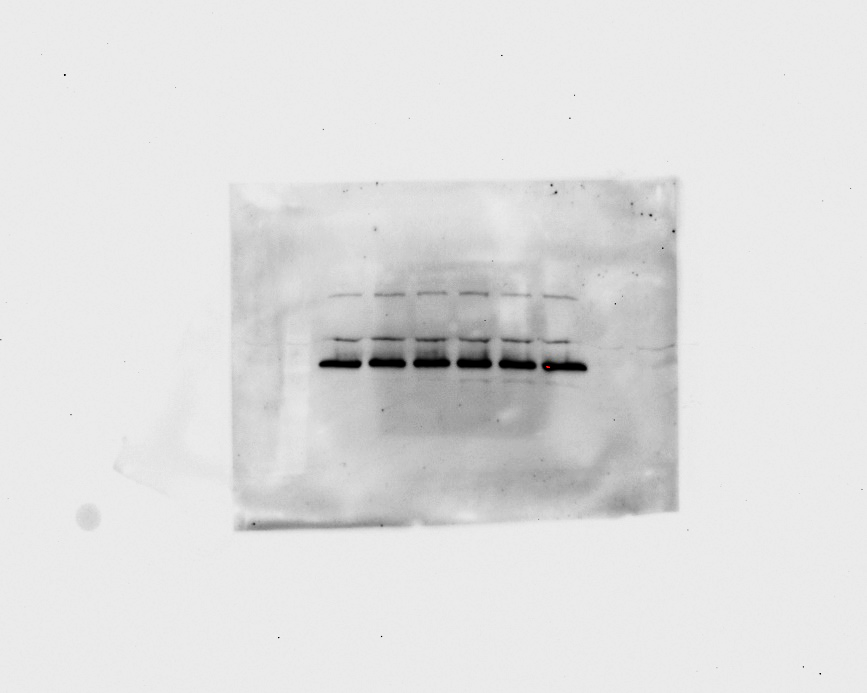


**GAPDH**

**37 kDa**

**Figure 7:** Raw data for agarose gel of Figure 6C


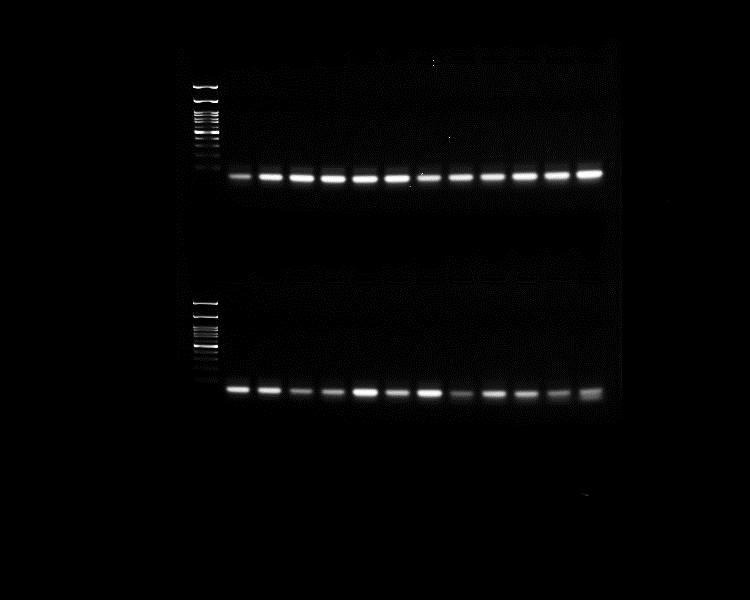


***Rac-1***

**84 bp**

18 h

24 h

2 h

***Rac-1***

***84 bp***

5 h


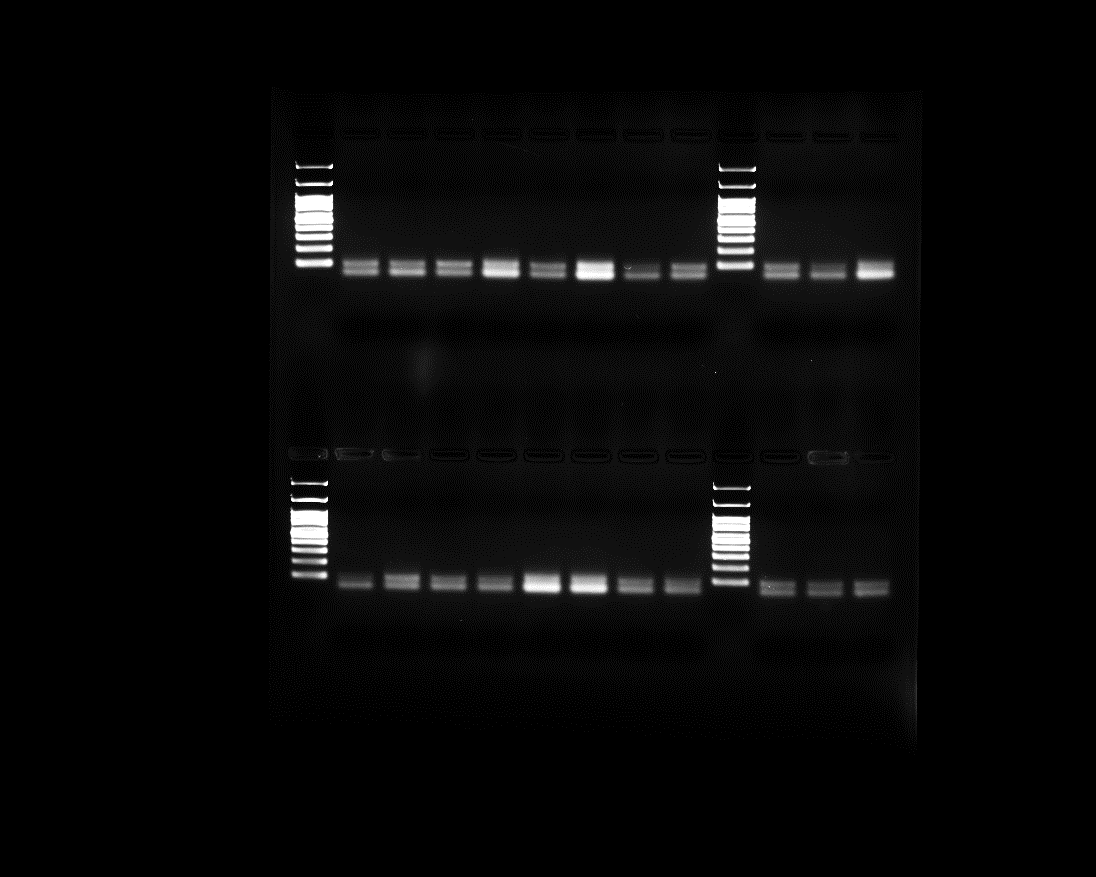


***Par-4***

***137 bp***

2 h

5 h

***Par-4***

***137 bp***


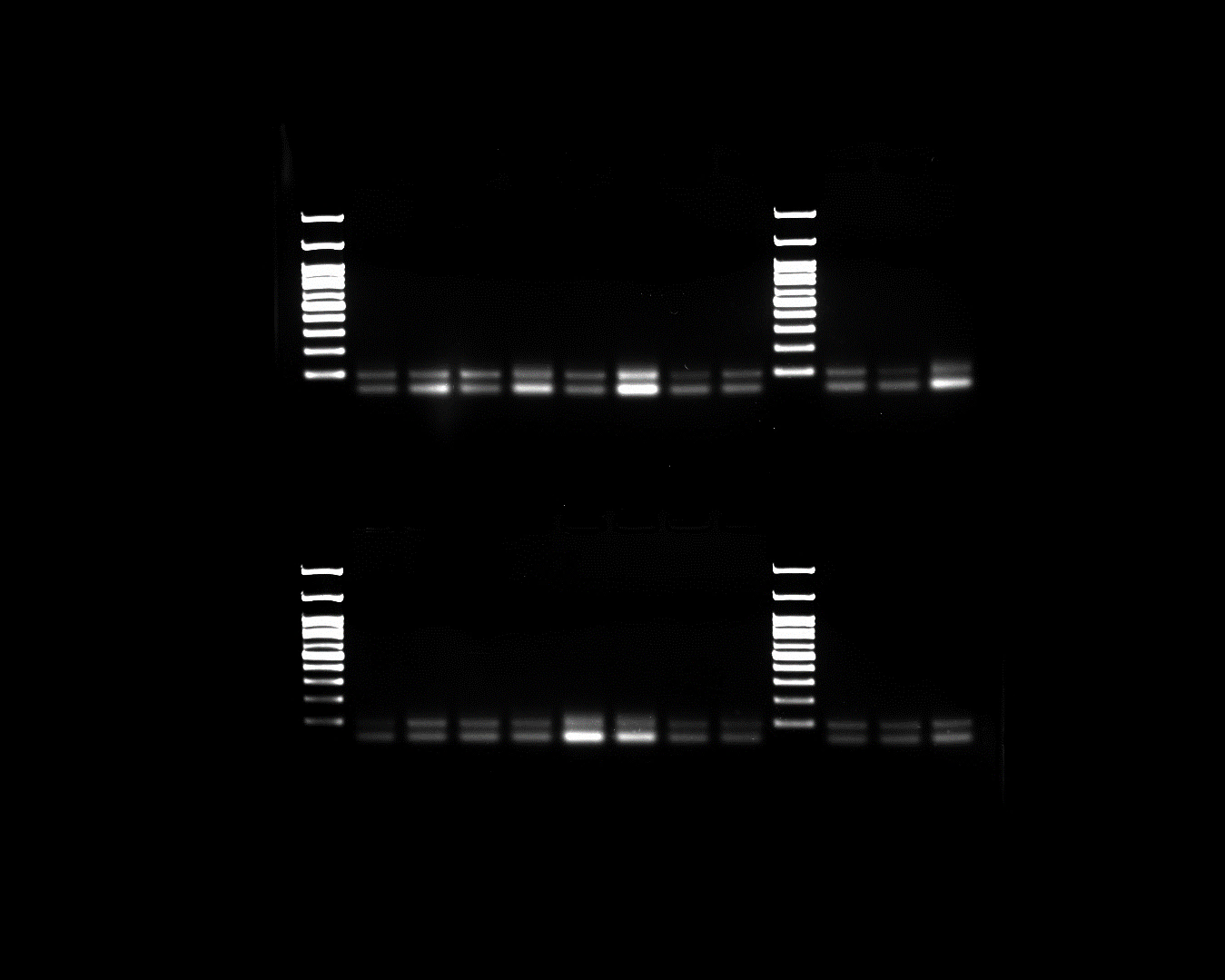

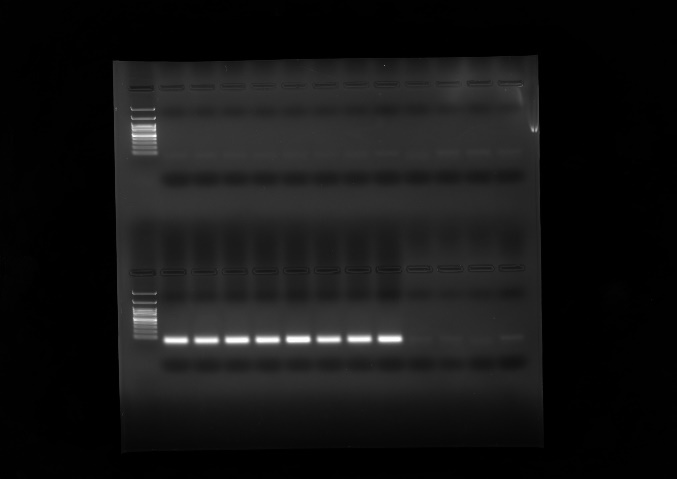

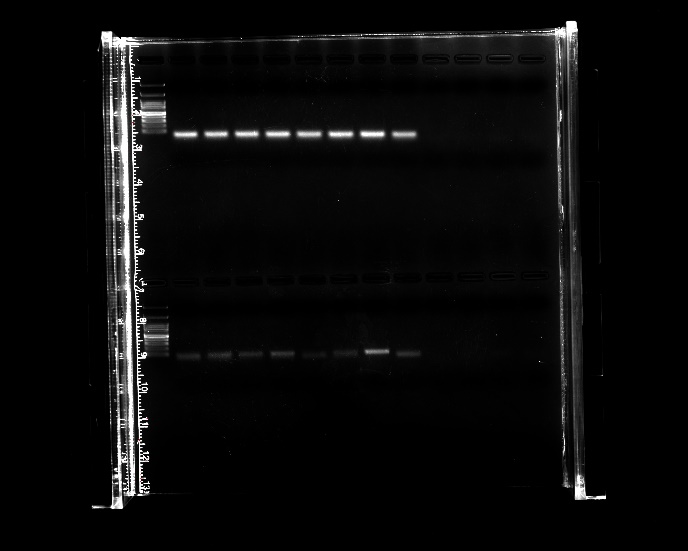


24 h

18 h

24 h

***Par-4***

**137 bp**

18 h

2 h

5 h

***gapdh***

***227 bp***

**Figure 8:** Raw data for agarose gel of Figure 6D


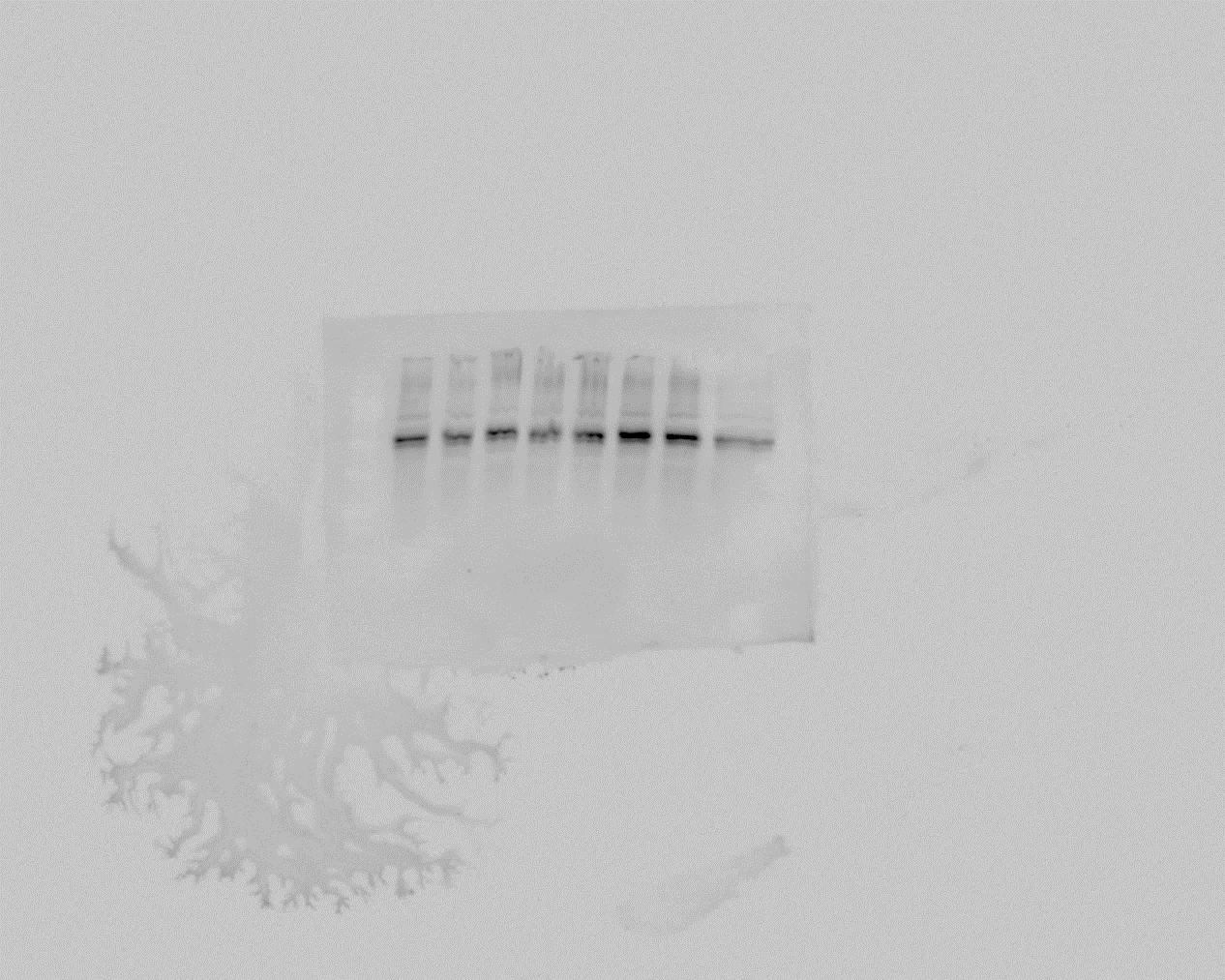


**Rac-1**

**20 kDa**

**Figure 8:** Raw data for agarose gel of Figure 6D


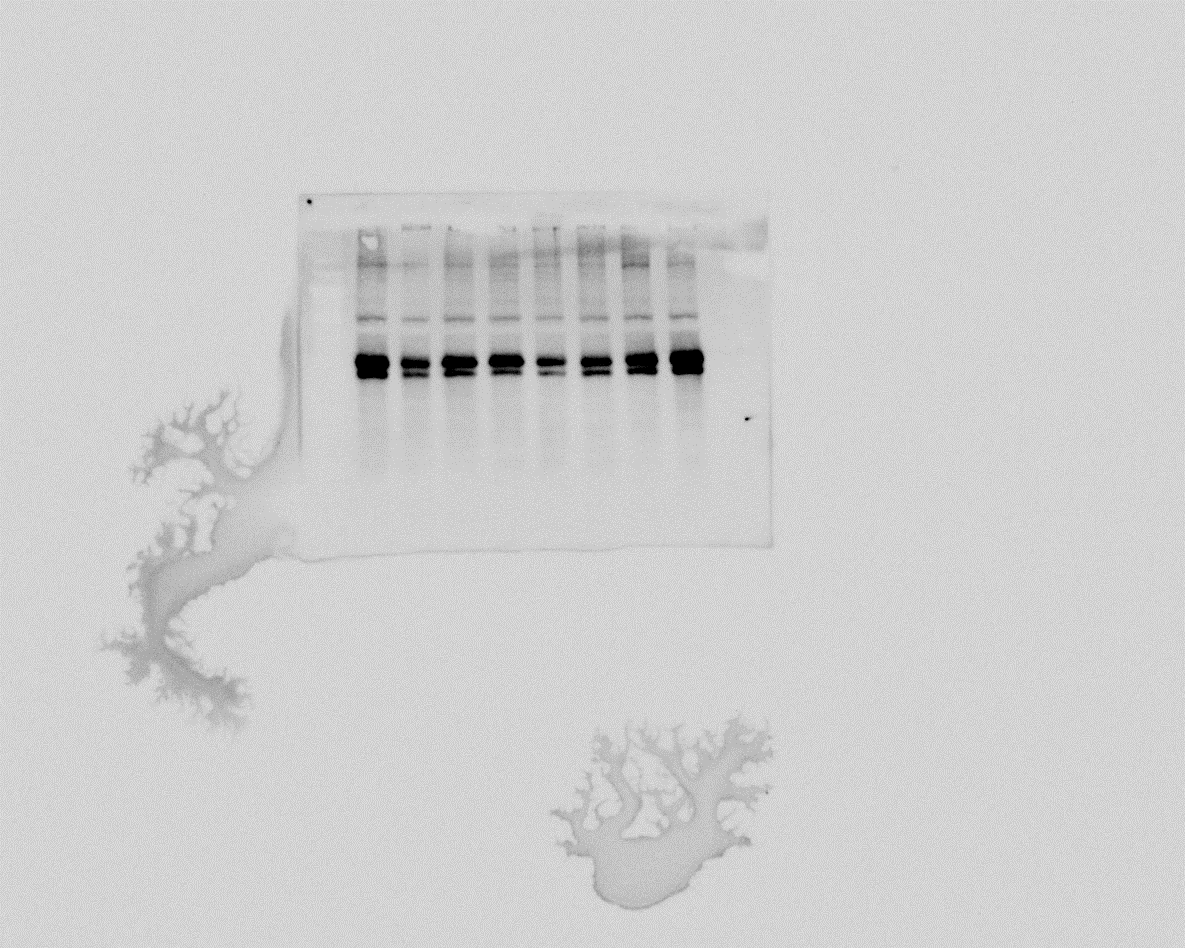


**Par-4**

**36 kDa**

**Figure 8:** Raw data for agarose gel of Figure 6D

**
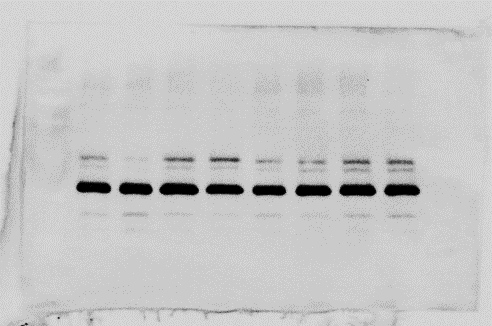
**

**GAPDH**

**37 kDa**

**Figure 9:** Raw data for agarose gel of Figure 6E and 6F


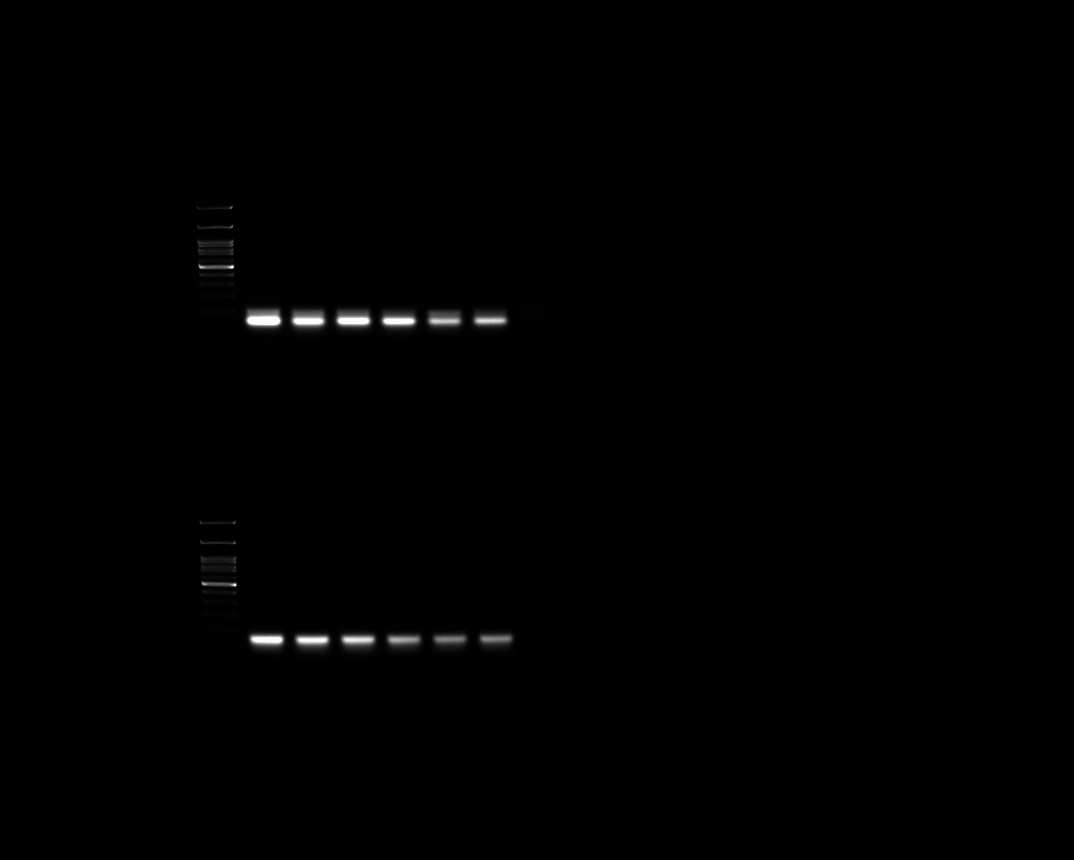


*Cdc42*

*101 bp*

5 h

24 h

*Cdc42*

*101 bp*


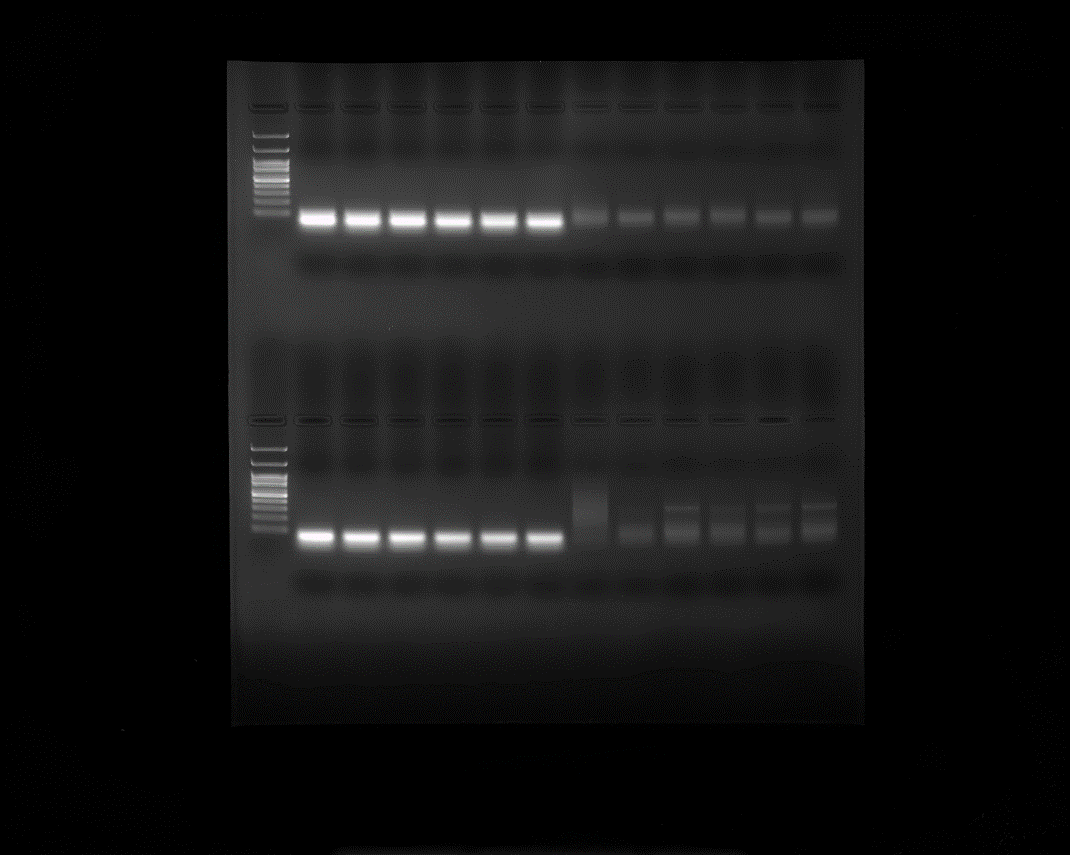


*gapdh*

*234 bp*

*gapdh*

*234 bp*

5 h

24 h


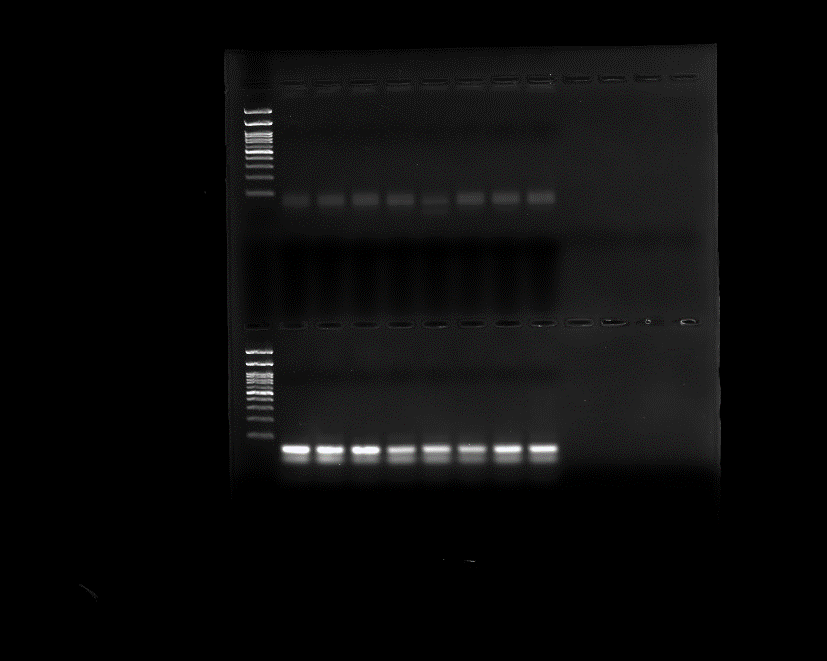


*VEGF*

*84 bp*


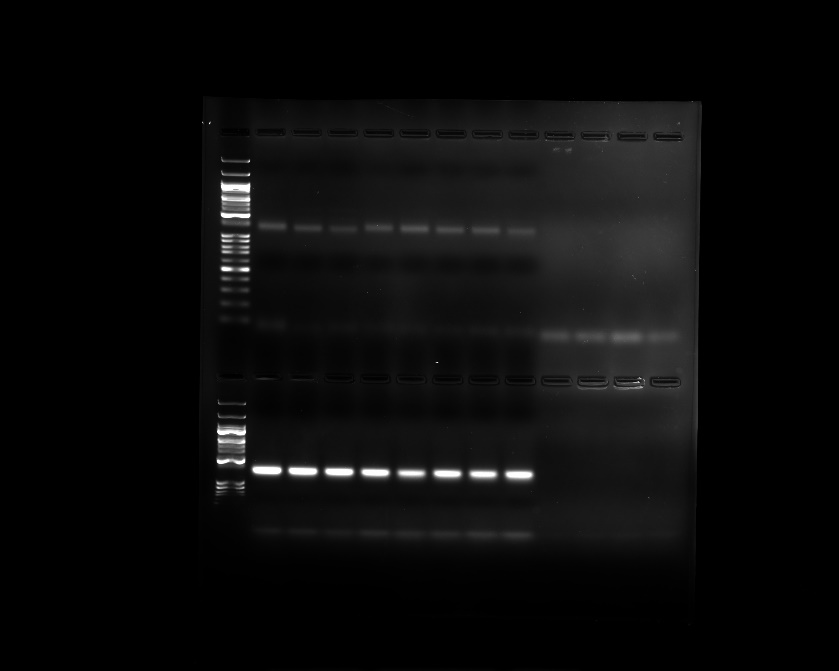


*gapdh*

*234 bp*
