# Supplementary material for: Ketorolac modulates Rac-1/HIF-1α/DDX3/β-catenin signalling via a tumor suppressor prostate apoptosis response-4 (Par-4) in renal cell carcinoma
Source: Sci Rep. 2023 Apr 6;13:5659. doi: 10.1038/s41598-023-32627-z (PMC10079967; doi:10.1038/s41598-023-32627-z)

**Additional File 3**

**Ketorolac modulates Rac-1/HIF-1α/DDX3/β-catenin signalling via a tumor suppressor Prostate apoptosis response-4 (Par-4) in renal cell carcinoma**

**Authors**

Vinay Sonawane^1†^, Jeevan Ghosalkar^1†^, Swati Achrekar^1^, Kalpana Joshi^1*^

**Affiliations**

^1^Cell Biology Division, Cipla Ltd., Vikhroli West, Mumbai - 400083, INDIA.

***Correspondence author**

Kalpana Joshi, Cell Biology Division, Cipla Ltd, LBS Marg, Vikhroli West, Mumbai – 400083, INDIA,

Tel: +91-22-25766326, Email: [kalpana.joshi@cipla.com](mailto:kalpana.joshi@cipla.com)

**^†^**Contributed equally to this work

This additional file contains (Figure 1, 2 and 3)

**Figure 1:** **Levels of secreted Par-4 post 24 h treatment with Ketorolac by A-498 cells.**


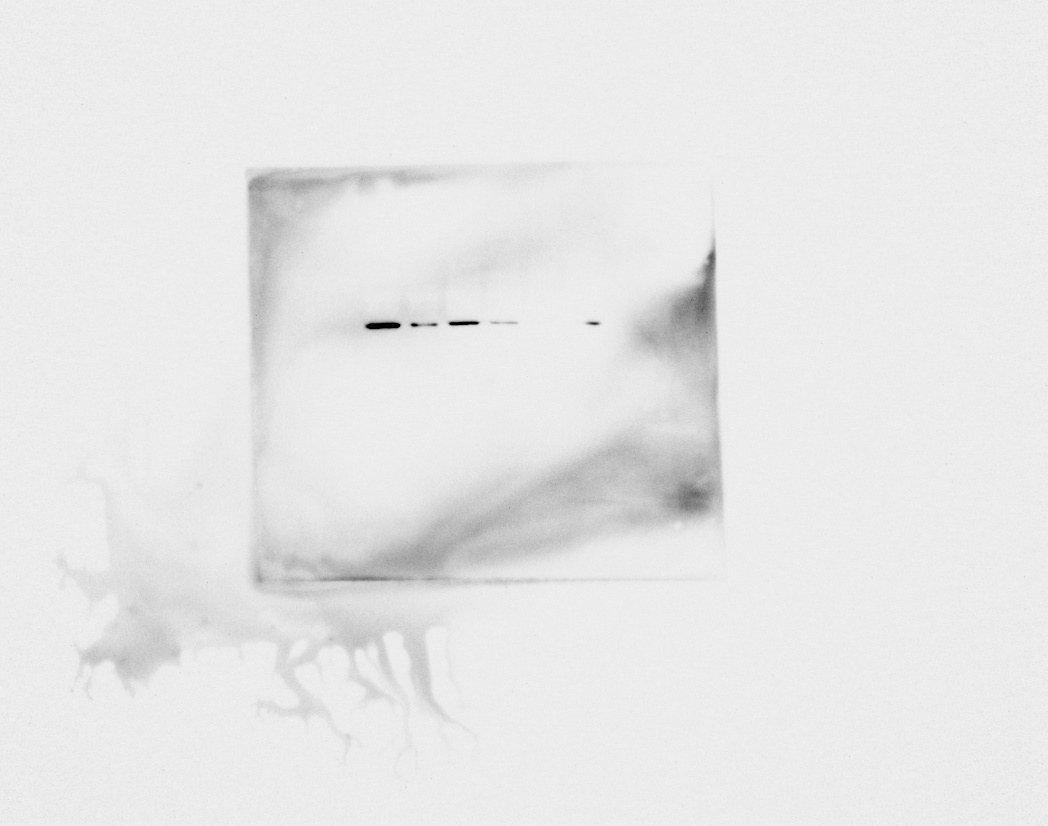


1. Control

2. Ketorolac – 1 mM

3. Ketorolac – 3 mM

4. Ketorolac – 7 mM

1 2 3 4

Secreted Par-4

41 KDa

Albumin stained with Ponceau was used as a loading control.

**Figure 2: Levels of secreted Par-4 post 24 h treatment with Ketorolac alone and in combination with Sunitinib by Atg cells.**


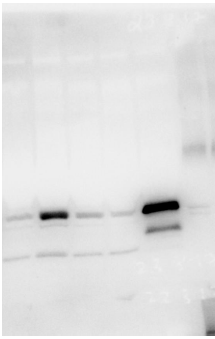


Par-4 secreted

41 KDa

Control

Sunitinib

Ketorolac

Ketorolac +

Sunitinib

Albumin stained with Coomassie was used as a loading control.

**Figure: 3. Wound healing assay for Ketorolac in 786-O**

Control

Ketorolac 3 mM

Ketorolac 4 mM

**
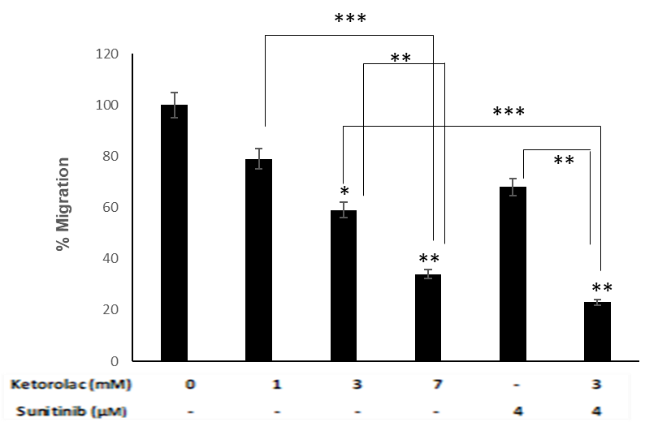

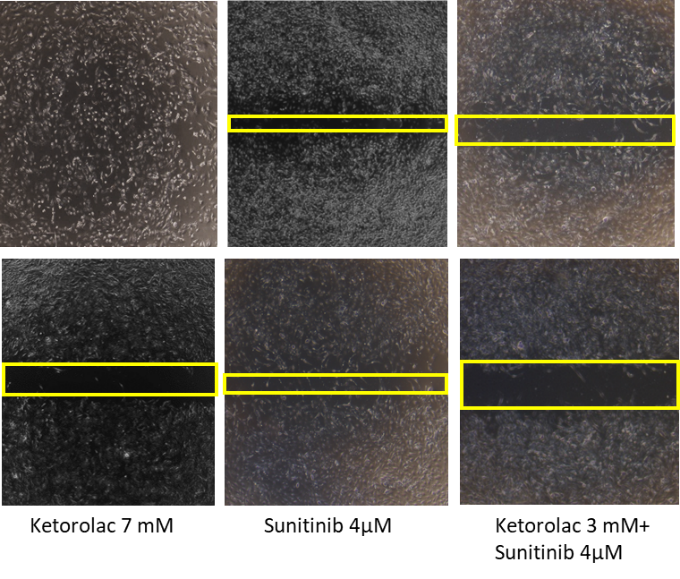
**

**a)**

**b)**

**Figure 3:** Wound healing assay for Ketorolac in 786-O. **a)** Wound-healing assay in 786-O treated with Ketorolac alone or in combination with Sunitinib. **b)** Histogram representing quantitation of images using Image J. Statistically significant difference between and within different groups was determined by one-way ANOVA and post hoc multiple variance by Tukey test (*p ≤ 0.05, **p≤ 0.01 and ***p≤ 0.001.

**Figure 4: The level of β-catenin and HIF-2α expression post 5 and 24 h treatment after silencing of *Par-4* and *Rac-1* independently**


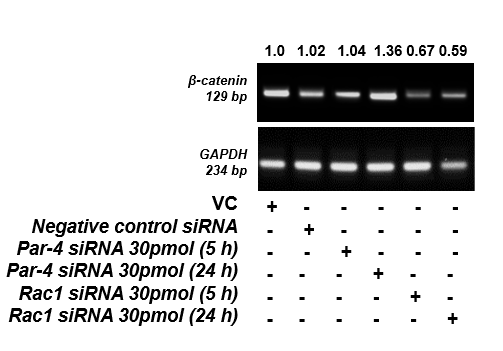


**b)**

**a)**


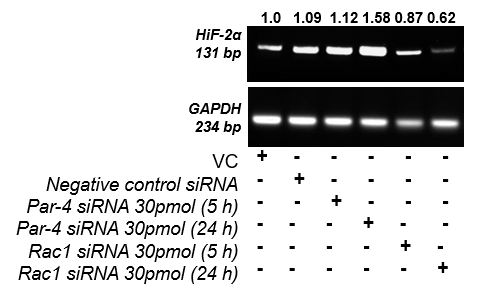


Figure 4: **a)** The level of β-catenin expression post 5 and 24 h after silencing Par-4 and Rac-1 independently. **b)** The level of HIF-2α expression post 5 and 24 h after silencing Par-4 and Rac-1 independently. gapdh served as a loading control. Numbers at top of the blots refer to the densitometric analysis of the immunoreactive bands and represent the fold change in gene and/or protein expression normalized to GAPDH.

**Full images for figure 4**

***β-catenin*  *HIF-2α***


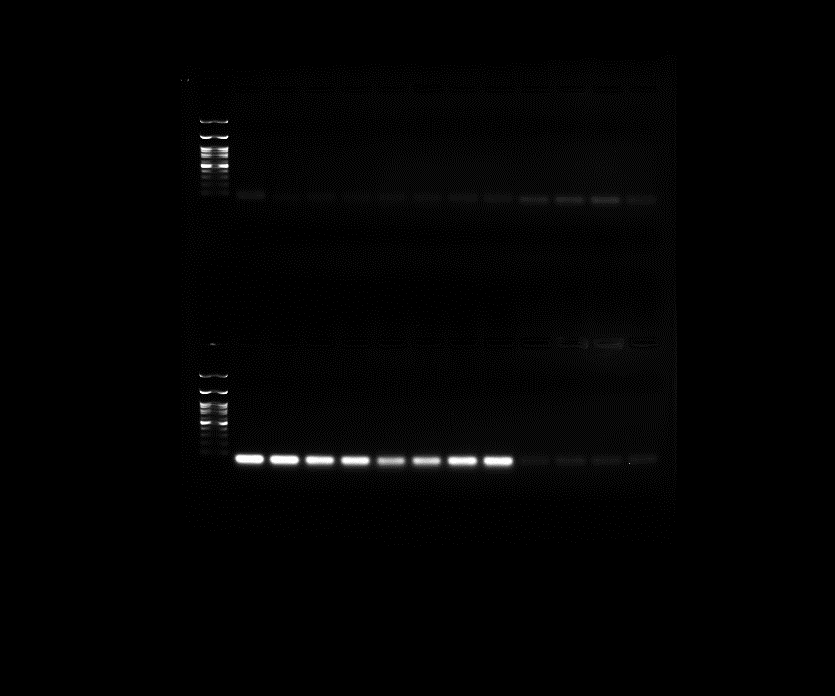

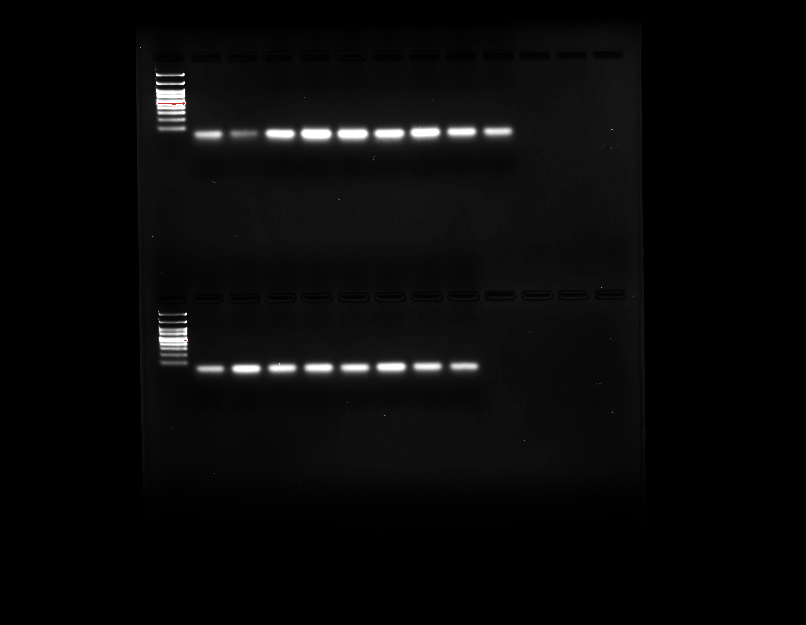


***GAPDH GAPDH***


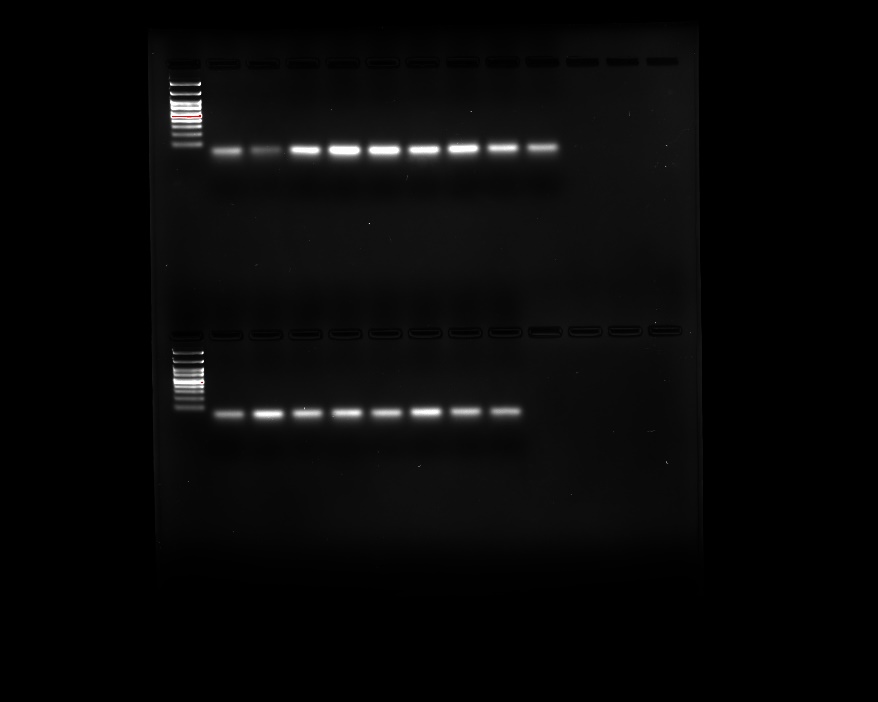

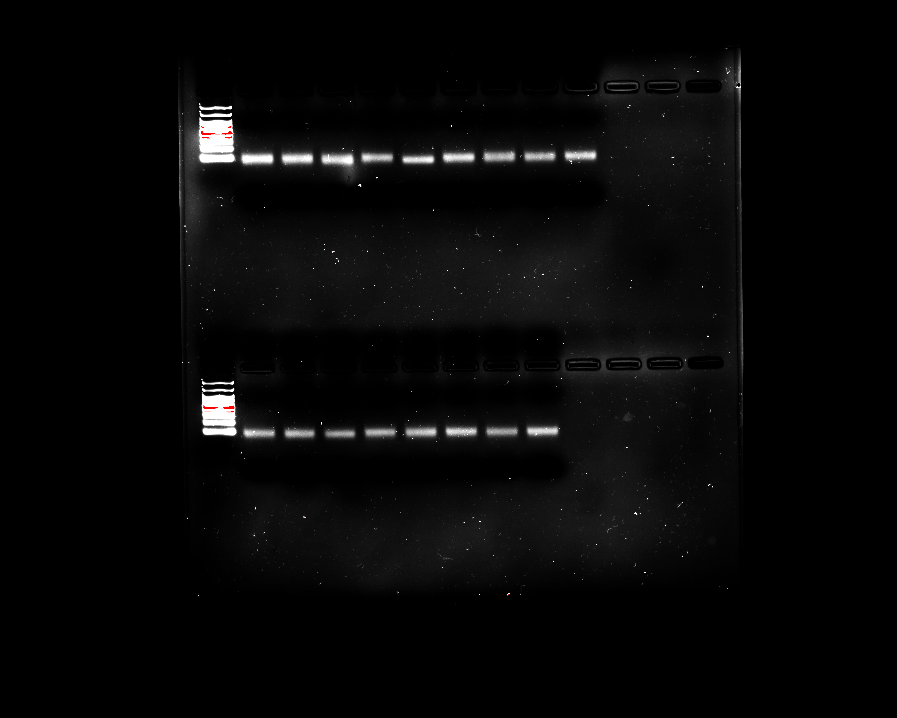

Supplement: Supplementary file 3 — Supplementary Information 3. [file 41598_2023_32627_MOESM3_ESM.docx]
